# Energy Deficiency‐Induced ATG4B Nuclear Translocation Inhibits PRMT1‐Mediated DNA Repair and Promotes Leukemia Progression

**DOI:** 10.1002/advs.202509838

**Published:** 2025-08-11

**Authors:** Zhenkun Wang, Xianli Zhang, Yuanyuan Zhou, Shuyi Lin, Yuting Fu, Haiping Liu, Qiongdan Gao, YanHong Xiao, Zhao Yin, Shuang Liu, Kexiu Huang, Suqi Deng, Juan Du, Hui Zeng, Jia Wang, Jinping Zheng, Qinghua Zhou, Tianfeng Chen, Xiaoling Gao, Zhenyu Ju, Bo Liu

**Affiliations:** ^1^ Key Laboratory of Regenerative Medicine of Ministry of Education Institute of Aging and Regenerative Medicine College of Life Science and Technology Jinan University Guangzhou 510632 China; ^2^ Shanxi Key Laboratory of Aging Mechanism Research and Translational Applications Changzhi Medical College Changzhi Shanxi 046000 China; ^3^ Biomedical Innovation Center Department of Clinical Laboratory The Sixth Affiliated Hospital Sun Yat‐sen University Guangzhou 510655 China; ^4^ Guangdong Second Provincial General Hospital Jinan university Guangzhou 510317 China; ^5^ Department of Hematology The First Affiliated Hospital of Jinan University Guangzhou 510630 China; ^6^ College of Life Science and Technology Jinan University Guangzhou 510632 China; ^7^ Department of Chemistry Jinan University Guangzhou 510632 China; ^8^ The clinical Laboratory Center Hainan General Hospital Hainan affiliated Hospital of Hainan Medical University Haikou 570311 China

**Keywords:** ATG4B, PRMT1, DNA repair, Energy metabolism, Leukemia

## Abstract

Metabolic alterations and genomic instability are the hallmark features of many cancers. However, the precise mechanisms underlying the intricate links among these processes remain largely unknown. Here, a molecular mechanism is presented that regulates the interplay between cellular energy metabolism and DNA repair. These findings demonstrate that during energy deficiency, ATG4B translocates from the cytoplasm to the nucleus and disrupts DNA repair by directly interacting with PRMT1. This interaction inhibits the PRMT1‐dependent methylation of MRE11, a key regulator of DNA repair, leading to genomic instability. Importantly, it is shown that ATG4B‐mediated DNA repair defects are significantly enhanced in patient‐derived acute myeloid leukemia (AML) cells and in mouse AML cells induced by MLLT3‐KMT2A overexpression. Inhibition of ATG4B enhanced PRMT1‐mediated DNA damage responses, suppressed cell proliferation, reduced the mutation burden, and prolonged survival in mice with MLLT3‐KMT2A‐induced AML and in those bearing AML patient‐derived xenografts. These findings revealed that energy deficiency compromises DNA repair through ATG4B nuclear translocation, and ATG4B inhibition enhances DNA repair in AML cells, alleviating the malignant evolution of AML.

## Graphical abstract

This study demonstrated that ATG4B translocates from the cytoplasm to the nucleus during energy deficiency. Nuclear ATG4B inhibits DNA repair by interacting with PRMT1. The nuclear ATG4B‐mediated DNA repair defect is significantly exacerbated within acute myeloid leukemia (AML) cells, promoting leukemia progression in an AML mouse model.

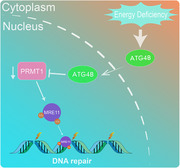

## 1 Introduction

Maintenance of Metabolic equilibrium and genomic stability are two critical biological processes within a cell. Dysregulation of these processes is frequently associated with various pathophysiological conditions such as aging and tumorigenesis. Although their individual significance is well established, emerging evidence suggests a potential interaction between cellular metabolism and genomic stability. Previous studies have demonstrated that cellular metabolism influences DNA repair and the DNA damage response via multiple pathways. Cellular metabolism regulates the epigenetic modification of chromatin, which is essential for modulating DNA remodeling during DNA repair processes.^[^ ^1^, ^2^, ^3^, ^4^ ^]^ Second, it controls the availability of nutrients required for de novo nucleotide synthesis, which is essential for DNA repair.^[^ ^5^, ^6^, ^7^ ^]^ Lastly, cellular metabolism may increase oxidative DNA damage by generating excessive reactive oxygen species (ROS) during metabolic processes, particularly during cellular energy metabolism.^[^ ^8^, ^9^, ^10^ ^]^ However, the precise molecular mechanisms that underlie the intricate link between cellular metabolism and genomic stability remain largely unknown.

DNA repair has long been believed to be a highly energy‐demanding process, consuming a significant amount of cellular ATP.^[^ ^11^, ^12^, ^13^ ^]^ However, the regulation of DNA repair by cellular energy metabolism has yet to be sufficiently explored. During radiation‐induced DNA repair, increased oxygen consumption and mitochondrial ATP generation have been observed to address the elevated cellular energy demands of DNA repair.^[^ ^11^ ^]^ Conversely, ROS generated by mitochondrial oxidative phosphorylation, fatty acid catabolism, and purine catabolism^[^ ^14^ ^]^ can damage DNA, thereby linking energy metabolism to DNA repair. Furthermore, an insufficient cellular energy supply results in impaired histone modifications in cancer cells, leading to reduced DNA repair.^[^ ^15^ ^]^ This highlights the significant role of energy deficiency in cancer progression through the inhibition of DNA repair. However, the molecular mechanisms by which cellular energy deficiency regulates DNA repair remain largely unknown.

Energy deficiency often induces autophagy; however, its role in DNA repair under energy deficiency remains unclear. Autophagy‐associated protein 4B (ATG4B) is a cysteine protease that cleaves cytoplasmic pro‐microtubule‐associated protein 1 light chain 3 (pro‐LC3) to generate LC3‐I, which can then be conjugated to phosphatidylethanolamine (PE) to form membrane‐bound LC3‐II.^[^ ^16^ ^]^ ATG4B also mediates the deconjugation of LC3 from PE, recycling LC3‐II back into LC3‐I.^[^ ^17^ ^]^ Furthermore, ATG4B is required for erythrocyte differentiation^[^ ^18^ ^]^ and otoconial development.^[^ ^19^ ^]^ Abnormal expression or mutations in ATG4B have been linked to tumorigenesis by affecting autophagy.^[^ ^20^, ^21^, ^22^, ^23^ ^]^ Notably, ATG4B regulates tumor cell proliferation independently of autophagic flux in colorectal cancer,^[^ ^24^ ^]^ suggesting a non‐canonical role of ATG4B beyond autophagy.

In this study, we demonstrated that ATG4B translocates from the cytoplasm to the nucleus during energy deficiency, independent of autophagy. Nuclear ATG4B inhibits DNA repair by interacting directly with PRMT1, which impairs PRMT1‐dependent methylation of MRE11, an important protein required for processing DNA breaks, thereby impairing DNA repair. Furthermore, we found that the nuclear ATG4B‐mediated DNA repair defect is significantly exacerbated within acute myeloid leukemia (AML) cells, promoting leukemia progression in an AML mouse model. These findings revealed a direct regulatory role of nuclear ATG4B in both DNA repair and leukemia progression.

## 2 Results

### 2.1 ATG4B Translocates from the Cytoplasm to the Nucleus During Starvation

**Figure 1:**
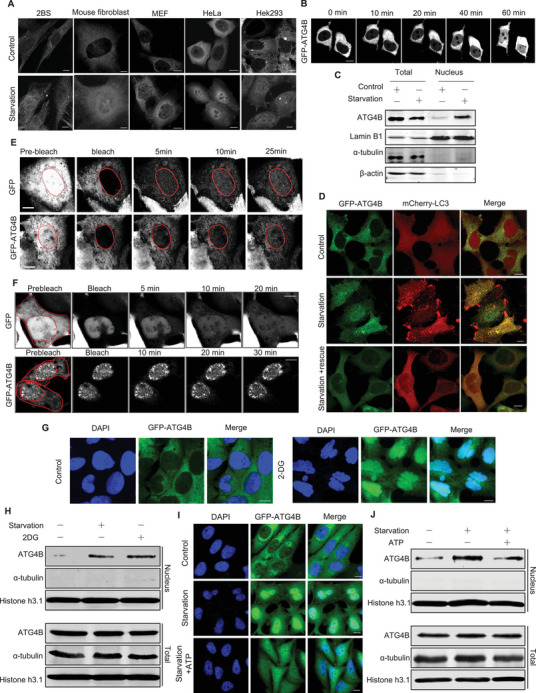
ATG4B translocates from the cytoplasm to the nucleus during energy deficiency A) Representative images of GFP‐ATG4B distribution in GFP‐ATG4B expressing human fibroblasts, mouse fibroblasts, MEF, HeLa, and HEK293 cells under fed (Control) or starvation conditions. Scale bar, 10 µm. B) Time‐lapse images of GFP‐ATG4B in GFP‐ATG4B‐expressing cells under starvation at indicated time point. Scale bar, 10 µm. C) Western blot analysis of nuclear ATG4B levels in HEK293 cells under starvation for 2 h, followed by nuclear extraction. D) Representative images of GFP‐ATG4B and mCherry‐LC3B distribution in HEK293 cells co‐expressing GFP‐ATG4B and mCherry‐LC3B under fed condition (Control), starvation for 2 h (Starvation), or starvation for 2 h followed by recovery under fed condition for 4 h (Starvation + Rescue). Scale bar, 10 µm. E) Representative images of GFP or GFP‐ATG4B distribution in cells under starvation, captured before or after photobleaching of nuclear GFP/GFP‐ATG4B signals at indicated time points. Scale bar, 5 µm. F) Representative images of GFP or GFP‐ATG4B distribution in cells under starvation, captured before or after photobleaching of cytoplasmic GFP/GFP‐ATG4B signals at indicated time points. Scale bar, 5 µm. G) Representative images of GFP‐ATG4B distribution in cells treated with or without 2 mm 2‐deoxyglucose (2‐DG) for 24 h. Scale bar, 10 µm. H) Western blot analysis of nuclear ATG4B levels in cells treated with or without 2 mM 2‐DG for 24 h, or starvation for 2 h. I) Representative images of GFP‐ATG4B distribution in cells treated with or without starvation for 2 h, or starvation supplemented with 20 µM ATP for 2 h. Scale bar, 10 µm. J) Western blot analysis of nuclear ATG4B levels in cells treated with or without starvation for 2 h, or starvation supplemented with 20  µM ATP for 2 h.

To investigate the role of ATG4B in cellular metabolism, we examined its subcellular localization in fed and starved cells. In various cell lines, including primary mouse fibroblasts, 2BS, MEF, HeLa, and HEK293 cells, ATG4B in fed cells (control) primarily resides in the cytoplasm. In starved cells, ATG4B showed typical nucleocytoplasmic localization, with a strong distribution in the nucleus (**Figure** **1**A). This indicated the translocation of ATG4B from the cytoplasm to the nucleus under starvation conditions. Next, nuclear translocation of ATG4B was monitored using time‐lapse confocal microscopy in HEK293 cells expressing GFP‐ATG4B. The results showed a gradual increase in ATG4B levels in the nucleus over time during starvation, confirming the continuous recruitment of ATG4B to the nucleus (Figure 1B). This nuclear translocation of ATG4B in starved cells was further validated by cell fractionation, which showed that endogenous ATG4B was enriched in the nuclear fraction (Figure 1C). Interestingly, the starvation‐induced nuclear translocation of ATG4B was reversed when starved cells were replenished with a nutrient‐rich medium for 0.5–4 h (Figure 1D; Figure S1A, Supporting Information).

Given the continuous recruitment of ATG4B to the nucleus during starvation, we sought to determine whether ATG4B undergoes consistent cycling in and out of the nucleus or becomes trapped. To investigate how ATG4B redistributes into the nucleus during starvation, we performed fluorescence recovery after photobleaching on the selectively nuclear (Figure 1E) or cytoplasmic pools (Figure 1F) of GFP‐ATG4B to monitor the dynamics of GFP‐ATG4B in the nucleus. In starved GFP‐expressing cells (as a control), after photobleaching the nuclear (Figure 1E) or cytoplasmic pools (Figure 1F) of GFP fluorescence, the GFP fluorescence signal quickly recovered from the unbleached areas, restoring nucleocytoplasmic localization. This suggests a constant exchange of GFP protein between the nucleus and cytoplasm (Figure 1E,F). In starved GFP‐ATG4B‐expressing cells, rapid recovery of GFP‐ATG4B fluorescence from the cytoplasm was observed after photobleaching of the fluorescence signal in the nucleus (Figure 1E). However, after bleaching of the GFP‐ATG4B fluorescence in the cytoplasmic pool, no recovery of GFP‐ATG4B fluorescence was observed (Figure 1F). This suggests that under starvation, ATG4B is trapped in the nucleus, possibly because of factors that prevent or hinder its return to the cytoplasm.

### 2.2 Energy Deficiency Results in the Nuclear Translocation of ATG4B

Starvation affects many intracellular processes, including protein synthesis, DNA damage, and energy metabolism. We investigated the factors contributing to the nuclear translocation of ATG4B. Neither the inhibition of protein synthesis by tunicamycin nor irradiation‐induced DNA damage resulted in the redistribution of ATG4B into the nucleus (Figure S1B–D, Supporting Information). Interestingly, significant nuclear translocation of ATG4B was observed in cells treated with 2‐deoxyglucose (2‐DG), which inhibits cellular ATP generation (Figure 1G,H). Conversely, the addition of ATP significantly inhibited the nuclear translocation of ATG4B during starvation (Figure 1I,J). Collectively, these data suggested that energy deficiency was responsible for the nuclear translocation of ATG4B.

### 2.3 Nuclear Translocation of ATG4B is Independent of Autophagy

Considering the canonical role of ATG4B in autophagy, we investigated whether nuclear translocation of ATG4B is required for autophagy. We induced autophagy via Torin1 treatment and analyzed the localization of ATG4B. Immunofluorescence and cell fractionation experiments showed that ATG4B predominantly resides in the cytoplasm during Torin1‐induced autophagy (Figure S2A,B, Supporting Information). Furthermore, the inhibition of autophagy by the AMPK inhibitor, Compound C, did not affect the nuclear translocation of ATG4B during starvation (Figure S2C,D, Supporting Information). Additionally, in both wild‐type and endogenous ATG4B knockout cells, mCherry‐LC3 was coexpressed with an ATG4B construct containing a nuclear export signal (NES‐ATG4B) that inhibited ATG4B nuclear translocation. Autophagy was successfully induced in cells under starvation conditions, as evidenced by the significantly enhanced LC3‐positive puncta (Figure S2E,F, Supporting Information). These findings suggest that nuclear translocation of ATG4B plays a role independent of its well‐characterized function in autophagy.

### 2.4 Nuclear Translocation of ATG4B Increases DNA Damage

**Figure 2:**
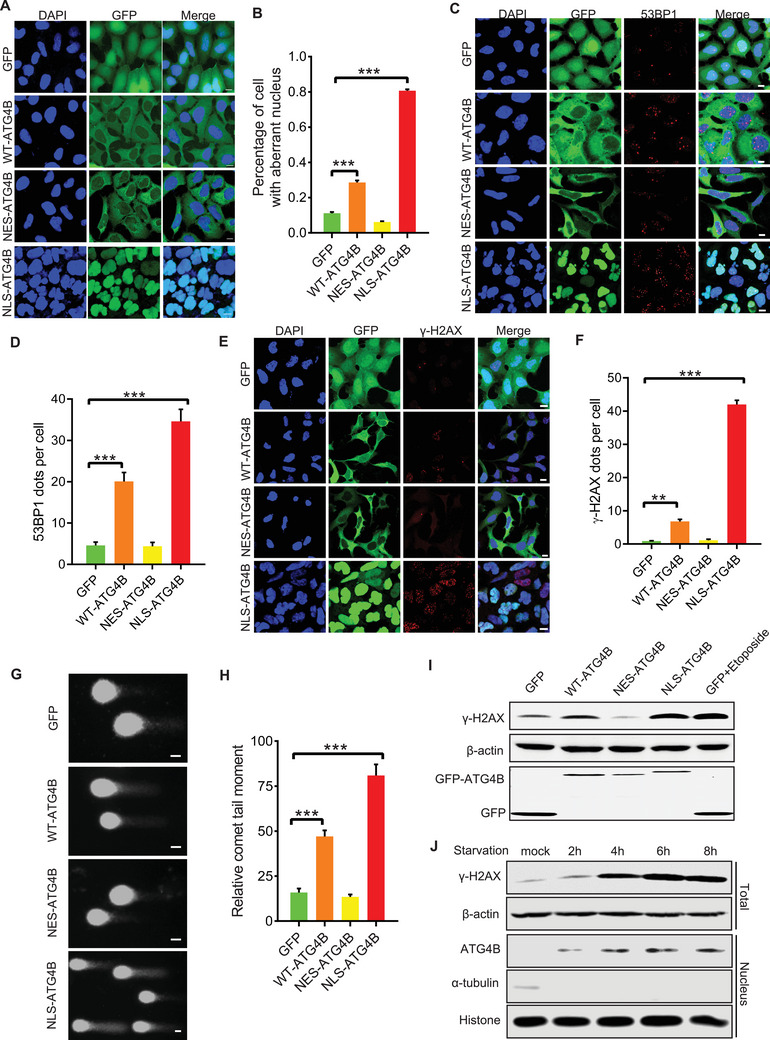
Nuclear translocation of ATG4B increases DNA damage A,B) Representative images and quantification of aberrant nuclei in cells overexpressing GFP, GFP‐WT‐ATG4B, GFP‐NES‐ATG4B, or GFP‐NLS‐ATG4B (n ≥ 10 randomly selected scope). Scale bar, 10 µm. C,D) Representative images and quantification of 53BP1 foci in cells overexpressing GFP, GFP‐WT‐ATG4B, GFP‐NES‐ATG4B, or GFP‐NLS‐ATG4B (n ≥ 50 cells). Scale bar, 10 µm. E,F) Representative images and quantification of γ‐H2AX foci in cells overexpressing GFP, GFP‐WT‐ATG4B, GFP‐NES‐ATG4B, or GFP‐NLS‐ATG4B (n ≥ 50 cells). Scale bar, 10 µm. G,H) Representative images and quantification of alkaline comet assays in cells overexpressing GFP, GFP‐WT‐ATG4B, GFP‐NES‐ATG4B, or GFP‐NLS‐ATG4B (n ≥ 50 comet tails). Scale bar, 20 µm. I) Western blot analysis of γ‐H2AX levels in cells overexpressing GFP, GFP‐WT‐ATG4B, GFP‐NES‐ATG4B, or GFP‐NLS‐ATG4B. J) Western blot analysis of γ‐H2AX levels and nuclear ATG4B levels after starvation for the indicated time in cells overexpressing GFP‐WT‐ATG4B. All the statistical data are represented as mean ± SEM. Statistical analyses were performed using two‐tailed unpaired *t*‐tests. ^**^ *p*< 0.01, ^***^ *p*< 0.001.

To further investigate the role of ATG4B in the nucleus, we constructed mCherry‐LC3 cell lines stably expressing GFP, GFP‐tagged WT‐ATG4B, NES‐ATG4B, or NLS‐ATG4B (with the latter containing a nuclear localization signal (NLS)). These cell lines were referred to as GFP‐cells, WT‐ATG4B‐cells, NES‐ATG4B‐cells, and NLS‐ATG4B‐cells (**Figure** **2**A). Intriguingly, we found that, in contrast to GFP‐ and NES‐ATG4B‐cells, a significant portion of NLS‐ATG4B‐cells exhibited aberrant nuclei, while WT‐ATG4B‐cells displayed only slight nuclear abnormalities (Figure 2A,B). These findings suggest that nuclear ATG4B is associated with increased DNA damage. To further confirm the increase in DNA damage associated with nuclear ATG4B, we examined the number of γ‐H2AX and 53BP1 foci, commonly recognized as hallmarks of DNA damage, in GFP‐, WT‐ATG4B‐, NES‐ATG4B‐, or NLS‐ATG4B‐cells. We found that, in contrast to GFP‐ and NES‐ATG4B‐cells, γ‐H2AX and 53BP1 foci were significantly increased in NLS‐ATG4B‐cells and slightly elevated in WT‐ATG4B‐cells (Figure 2C–F). Meanwhile, increased levels of γ‐H2AX and DNA breaks were consistently observed in both the NLS‐ATG4B‐cells and WT‐ATG4B‐cells, as determined by western blotting and the comet assay (Figure 2G–I). In addition, with the increase of ATG4B protein in the nucleus, a gradually increased γ‐H2AX protein levels were observed in HEK293 cells under starvation (Figure 2J). Collectively, these data suggest that the nuclear translocation of ATG4B causes DNA damage.

### 2.5 Nuclear ATG4B Impairs DNA Repair

**Figure 3:**
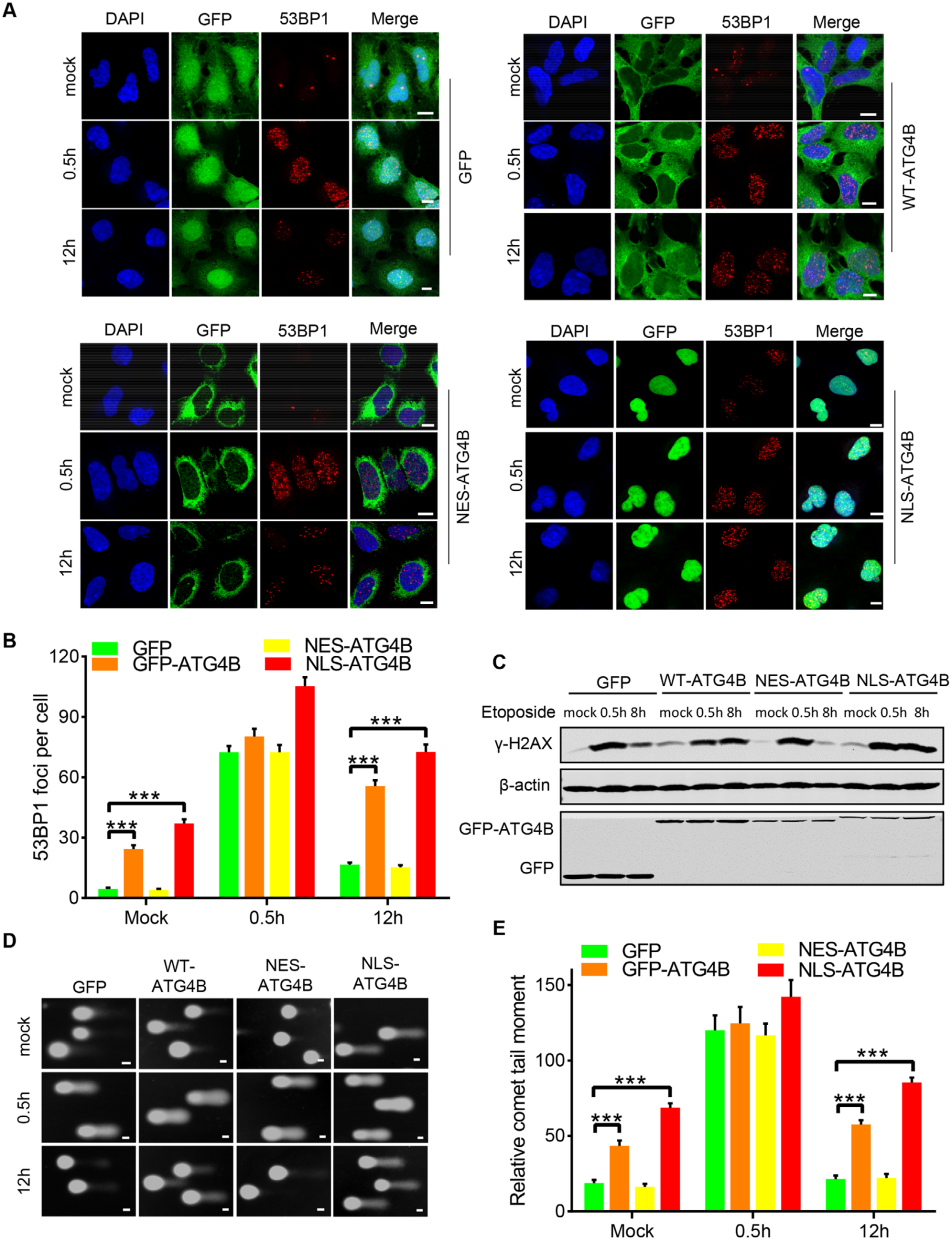
Nuclear ATG4B impairs DNA repair. A,B) Representative images and quantification of 53BP1 foci in cells overexpressing GFP, GFP‐WT‐ATG4B, GFP‐NES‐ATG4B, or GFP‐NLS‐ATG4B after treatment with 5 µM etoposide for 2 h followed by recovery for the indicated time (0.5 and 12 h) (n ≥ 50 cells). Scale bar, 10 µm. C) Western blot analysis of γ‐H2AX levels in cells respectively overexpressing GFP, GFP‐WT‐ATG4B, GFP‐NES‐ATG4B, or GFP‐NLS‐ATG4B after treatment with 5 µM etoposide for 2 h followed by recovery for the indicated time (0.5 and 8 h). D,E) Representative images and quantification of alkaline comet assays in cells treated as in (A). Scale bar, 20 µm. (n ≥ 50 comet tails). All the statistical data are represented as mean ± SEM. Statistical analyses were performed using two‐tailed unpaired t‐tests. ^***^ *p*<0.001.

To investigate the mechanism by which nuclear ATG4B increased DNA damage, we evaluated the DNA repair capacity of GFP‐, WT‐ATG4B‐, NES‐ATG4B‐, and NLS‐ATG4B‐cells. First, the cells were pretreated with the DNA‐damaging agent etoposide, an antagonist of DNA topoisomerase II, for 2 h. Following this treatment, we examined the formation of 53BP1 foci and the levels of γ‐H2AX protein. Similar increases in 53BP1 foci and γ‐H2AX protein levels were detected in the cells 0.5 h after removal of etoposide (**Figure** **3**A–C), suggesting that DNA damage was induced by etoposide. Eight or twelve hours after removal of etoposide, a marked decrease in 53BP1 foci and γ‐H2AX protein levels was observed in GFP‐ and NES‐ATG4B‐cells (Figure 3A–C), indicating successful DNA repair in these cells. However, in WT‐ATG4B‐cells and particularly in NLS‐ATG4B‐cells, the DNA repair capacity was significantly impaired, as demonstrated by the markedly retained 53BP1 foci and γ‐H2AX protein in these cells (Figure 3A–C). Additionally, impairment of DNA break repair following etoposide treatment was consistently observed in WT‐ATG4B and NLS‐ATG4B‐cells, as demonstrated by the comet assay (Figure 3D,E). Collectively, these data suggest that nuclear ATG4B impairs the DNA repair capacity.

### 2.6 Nuclear ATG4B Interacts with PRMT1 to Regulate MRE11 Methylation

**Figure 4:**
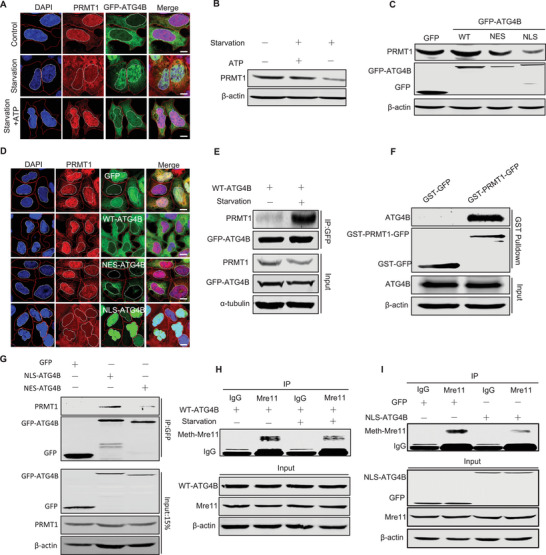
Nuclear ATG4B interacts with PRMT1 A) Representative images of nuclear GFP‐ATG4B and PRMT1 levels in cells treated with or without starvation for 2 h, or starvation supplemented with 20 µM ATP for 2 h. Scale bar, 10 µm. B) Western blot analysis of PRMT1 levels in cells treated with or without starvation for 2 h, or starvation supplemented with 20 µM ATP for 2 h. C) Representative images of nuclear PRMT1 levels in cells overexpressing GFP, GFP‐WT‐ATG4B, GFP‐NES‐ATG4B, or GFP‐NLS‐ATG4B. Scale bar, 10 µm. D) Western blot analysis of PRMT1 levels in cells overexpressing GFP, GFP‐WT‐ATG4B, GFP‐NES‐ATG4B, or GFP‐NLS‐ATG4B. E) Western blot analysis of PRMT1 co‐precipitated with GFP‐tagged ATG4B proteins. Immune complexes were obtained from lysates of GFP ‐WT‐ATG4B cells treated with or without starvation for 4 h. F) Western blot analysis of ATG4B co‐precipitated with in vitro purified GST‐PRMT1‐GFP or GST‐GFP. G) Western blot analysis of PRMT1 co‐precipitated with GFP‐tagged ATG4B proteins. Immune complexes were obtained from lysates of cells overexpressing GFP, GFP‐NES‐ATG4B, or GFP‐NLS‐ATG4B. H, I) MRE11 was immunoprecipitated from lysates of cells overexpressing GFP‐WT‐ATG4B treated with or without starvation for 4 h H) or cells overexpressing GFP or GFP‐NLS‐ATG4B I), followed by western blot analysis of the methylation levels of MRE11.

To investigate how ATG4B inhibits DNA repair, we screened for ATG4B‐interacting proteins in cells stably expressing Flag‐ATG4B, using co‐immunoprecipitation combined with mass spectrometry (Figure S3A, Supporting Information). Among the DNA repair‐related proteins that co‐immunoprecipitated with ATG4B, we focused on the most abundant protein, PRMT1.^[^ ^25^, ^26^ ^]^ Interestingly, we found that the levels of PRMT1 protein, particularly in the nucleus, were significantly decreased under starvation conditions. This decrease was associated with an increase in ATG4B nuclear translocation (**Figure** **4**A,B). This starvation‐induced PRMT1 reduction was markedly ameliorated by ATP supplementation and was inversely correlated with the suppression of ATG4B nuclear translocation (Figure 4A,B). In addition, a significant decrease in PRMT1 protein was observed in NLS‐ATG4B‐cells, but not in cells expressing C74S‐ATG4B, an inactive ATG4B mutant lacking protease activity (Figure 4C,D; Figure S3B,C, Supporting Information). These data suggested that nuclear ATG4B regulates PRMT1.

To further verify the role of ATG4B in the regulation of PRMT1, we examined the interaction between ATG4B and PRMT1 using co‐immunoprecipitation analysis. A significant interaction between ATG4B and PRMT1 was observed under starvation conditions (Figure 4E). An in vitro GST pull‐down assay confirmed the interaction between ATG4B and PRMT1, as GST‐PRMT1 significantly pulled down ATG4B from cell lysates (Figure 4F). Furthermore, significant co‐immunoprecipitation of PRMT1 with GFP‐NLS‐ATG4B was observed, whereas only a small amount of PRMT1 was co‐immunoprecipitated with GFP‐NES‐ATG4B (Figure 4G). These data indicate an interaction between PRMT1 and nuclear ATG4B. Given the protease role of ATG4B in autophagy, we wondered whether a directly proteolytic cleaves PRMT1 by ATG4B. Therefore, proteolytic cleavage of recombinant ATG4B and PRMT1 was performed in vitro. The results showed ATG4B cleaved PRMT1 in a dose‐dependent manner; however, its cleavage efficiency was weaker than that of LC3B (Figure S3D,E, Supporting Information). These findings are consistent with previous reports showing that newly synthesized LC3B is rapidly cleaved by ATG4B^[^ ^27^ ^]^ and suggest a special regulation of ATG4B on PRMT1. PRMT1 is reported to regulate DNA repair by methylating MRE11, a key effector member of the MRN complex involved in DNA repair.^[^ ^28^ ^]^ Given the inhibition of DNA repair by nuclear ATG4B (Figure 3), we wondered whether MRE11 methylation was affected under starvation conditions. Consistently, we found that MRE11 methylation was significantly reduced in starved cells (Figure 4H). Furthermore, a marked suppression of methylated MRE11 was observed in NLS‐ATG4B‐cells (Figure 4I). These data suggested that nuclear ATG4B inhibits PRMT1 during DNA repair.

### 2.7 Nuclear ATG4B Inhibits DNA Repair via PRMT1

**Figure 5:**
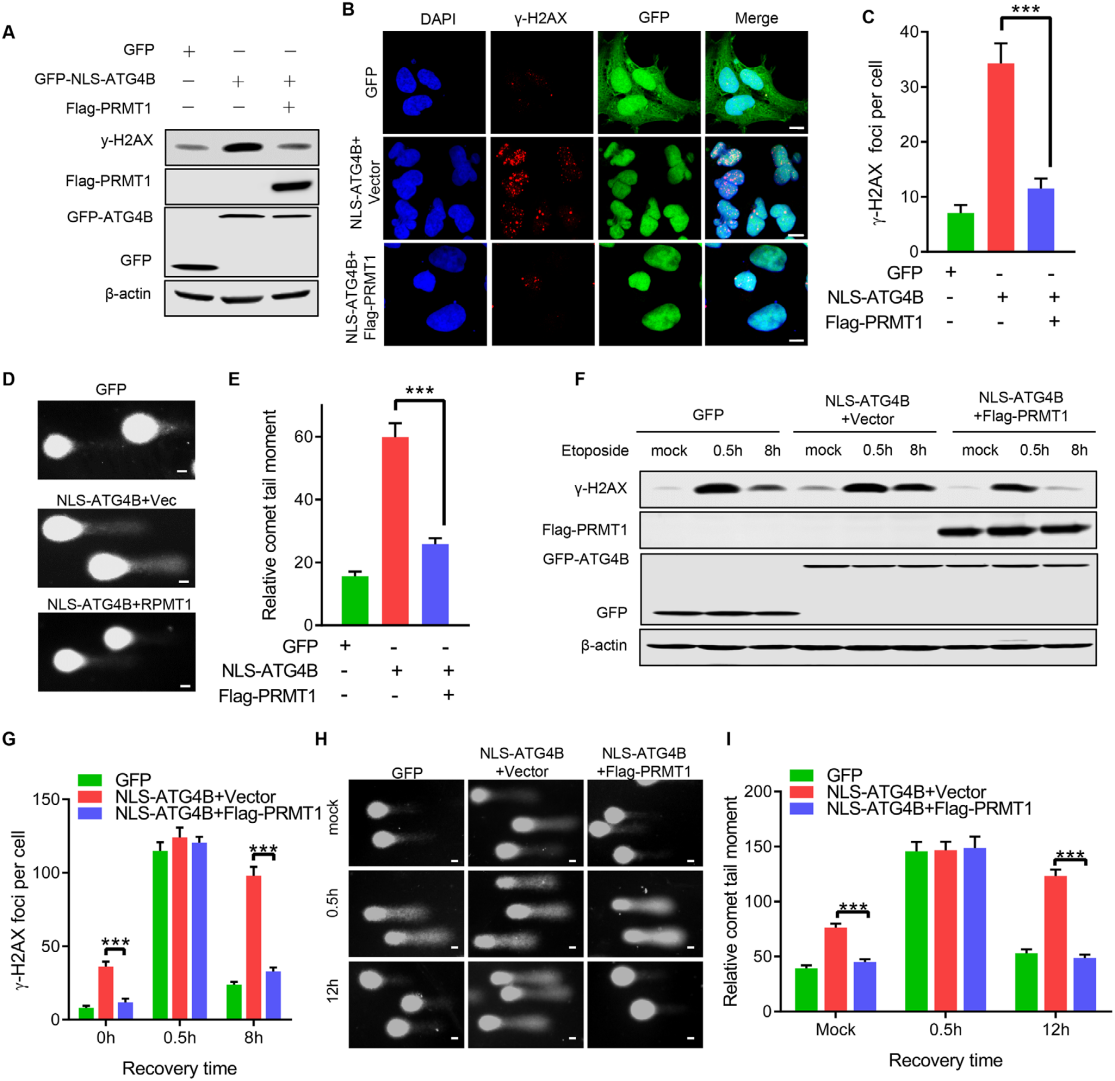
PRMT1 mediates the nuclear ATG4B‐impaired DNA repair. A) Western blot analysis of γ‐H2AX levels in cells overexpressing GFP, GFP‐NLS‐ATG4B, or Flag‐PRMT1 with GFP‐NLS‐ATG4B. B,C) Representative images and quantification of γ‐H2AX foci in cells overexpressing GFP, GFP‐NLS‐ATG4B, or Flag‐PRMT1 with GFP‐NLS‐ATG4B. (n ≥ 50 cells). Scale bar, 10 µm. D,E) Representative images and quantification of alkaline comet assays in cells overexpressing GFP, GFP‐NLS‐ATG4B, or Flag‐PRMT1 with GFP‐NLS‐ATG4B. (n ≥ 50 comet tails). Scale bar, 20 µm. F) Western blot analysis of γ‐H2AX levels in cells overexpressing GFP, GFP‐NLS‐ATG4B, or Flag‐PRMT1 with GFP‐NLS‐ATG4B after treatment with 5 µM etoposide for 2 h followed by recovery for the indicated time (0.5 and 8 h). G) Quantification of γ‐H2AX foci in cells treated as in (F) (n ≥ 50 cells). H,I) Representative images and quantification of alkaline comet assays in cells overexpressing GFP, GFP‐NLS‐ATG4B, or Flag‐PRMT1 with GFP‐NLS‐ATG4B after treatment with 5 µM etoposide for 2 h followed by recovery for the indicated time (0.5 and 12 h). Scale bar, 20 µm. All the statistical data are represented as mean ± SEM. Statistical analyses were performed using two‐tailed unpaired t‐tests. ^***^ *p*< 0.001.

To verify the role of PRMT1 in mediating nuclear ATG4B‐dependent impairment of DNA repair, we co‐expressed Flag‐tagged PRMT1 in NLS‐ATG4B‐cells and assessed the levels of DNA damage. We found that γ‐H2AX foci and protein levels were significantly reduced in NLS‐ATG4B‐cells co‐expressing Flag‐PRMT1 (**Figure** **5**A–C). Additionally, a significant reduction in DNA breaks was observed in NLS‐ATG4B‐cells co‐expressing Flag‐PRMT1, as demonstrated by the comet assay (Figure 5D,E).

To further confirm the role of PRMT1 in nuclear ATG4B‐impaired DNA repair, we assessed the DNA repair capacity of NLS‐ATG4B‐cells co‐expressing Flag‐PRMT1 after etoposide treatment. Overexpression of Flag‐PRMT1 led to a significant reduction in γ‐H2AX foci and protein levels in NLS‐ATG4B‐cells 8 h after drug removal (Figure 5F,G; Figure S4A–C, Supporting Information). The comet assay also revealed fewer DNA breaks in NLS‐ATG4B‐cells co‐expressing Flag‐PRMT1 12 h after drug washout (Figure 5H,I). In addition, a significant reduction in γ‐H2AX foci was observed in WT‐ATG4B‐cells co‐expressing Flag‐PRMT1 under starvation conditions (Figure S4D,E, Supporting Information). Collectively, these data indicated that increased PRMT1 expression enhanced the DNA repair capacity of NLS‐ATG4B‐cells.

### 2.8 Nuclear Translocation of ATG4B Contributes to Leukemia Progression in Mice

**Figure 6:**
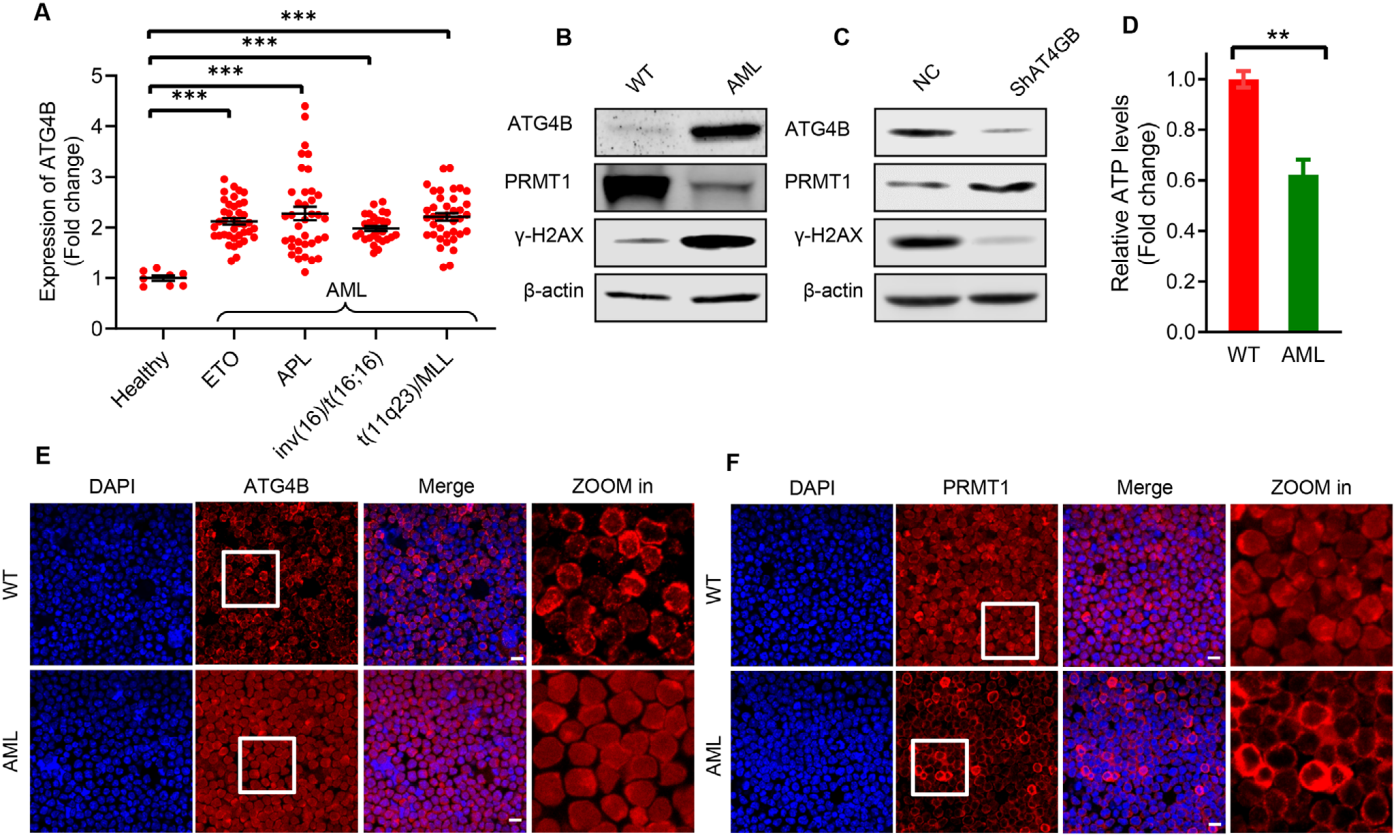
ATG4B is highly expressed in acute myeloid leukemia (AML) cell and translocates to the nucleus of leukemia cell. A) RNA‐seq data showing ATG4B expression levels in different genotypes of AML from the BloodSpot dataset. B) Western blot analysis of ATG4B, PRMT1, and γ‐H2AX levels in mice bone‐marrow‐derived WT or AML c‐kit^+^ cells. C) Western blot analysis of ATG4B, PRMT1, and γ‐H2AX levels in mice bone‐marrow‐derived AML cells expressing ATG4B‐targeted shRNA (shATG4B) or scrambled shRNA (shNC). D) Relative ATP levels in mice bone‐marrow‐derived WT or AML c‐kit^+^ cells. E,F) Representative images of ATG4B and PRMT1 distribution in mice bone‐marrow‐derived WT or AML c‐kit^+^ cells. Scale bar, 10 µm. All the statistical data are presented as mean ± SEM. Statistical analyses were performed using two‐tailed unpaired t‐tests. ^**^ *p*< 0.01, ^***^ *p*< 0.001.

ATG4B is involved in the tumorigenesis of multiple cancers, including osteosarcoma, breast cancer, and colorectal cancer.^[^ ^20^, ^21^, ^22^, ^23^, ^24^ ^]^ Given the critical role of DNA damage in cancer progression, we analyzed the expression levels of ATG4B in multiple cancers using publicly available datasets. We found that ATG4B exhibited significantly high expression levels in various acute myeloid leukemia (AML) with diverse molecular subgroups (e.g., MLL‐rearrangement, FLT3‐ITD mutation, and NPM1 mutation, etc.) (**Figure** **6**A; Figure S5A–C, Supporting Information). ATG4B showed increased chromatin accessibility in AML cells (Figure S5D, Supporting Information). Notably, patients with AML with high expression levels of ATG4B were statistically associated with poor survival and earlier relapse after treatment (Figure S5E–G, Supporting Information). These findings suggest an important role for ATG4B in leukemia progression.

To investigate the role of ATG4B in leukemia progression, we examined the protein levels of ATG4B in AML cells. Consistently, the results showed a significant increase in protein levels of ATG4B and γ‐H2AX in mouse AML cells induced by MLLT3‐KMT2A fusion gene (t(9;11)(p21.3;q23.3)) overexpression, accompanied by a corresponding decrease in PRMT1 protein levels (Figure 6B). ATG4B knockdown significantly increased PRMT1 protein levels and enhanced DNA repair in AML cells, as evidenced by a significant decrease in γ‐H2AX protein levels (Figure 6C). Notably, compared with WT cells, ATP levels in AML cells were significantly reduced (Figure 6D), and in starved WT‐hematopoietic stem/progenitor cells, ATG4B also showed a strong distribution in the nucleus (Figure S5H, Supporting Information). Furthermore, immunostaining revealed a significant increase in ATG4B and a decrease in PRMT1 levels in the nuclei of mouse AML cells induced by MLLT3‐KMT2A overexpression (Figure 6E,F). These data suggest an important role for nuclear ATG4B and PRMT1 in the progression of leukemia.

**Figure 7:**
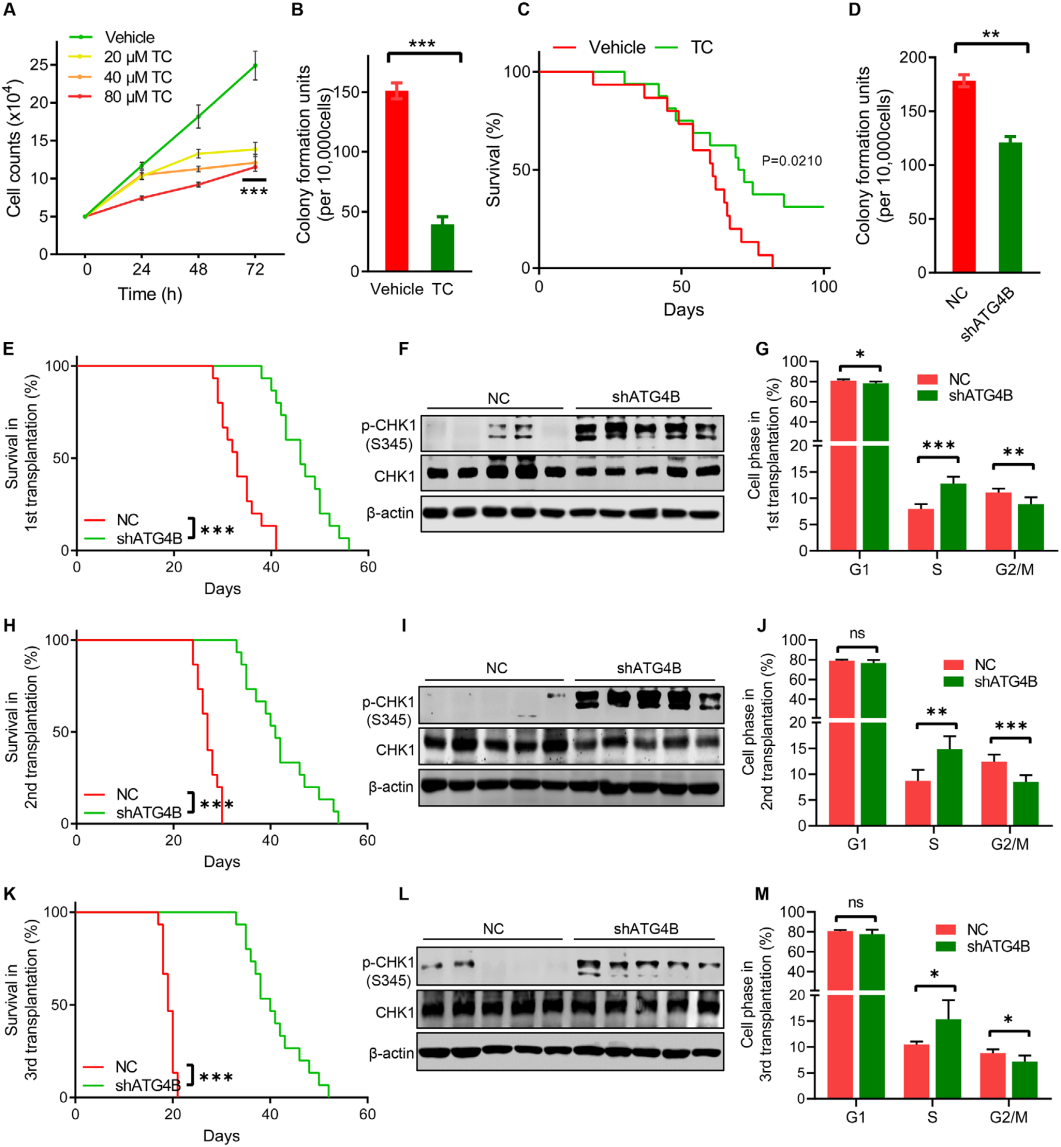
ATG4B inhibition suppresses leukemia progression in mice A) Mice bone‐marrow‐derived AML cells were seeded in a 24‐well plate (5 × 10^4^ cells per well, n = 5), treated with or without the indicated concentration of tioconazole (TC), and cell numbers were counted every 24 h. B) Colony‐forming assay of mice bone marrow derived AML cells treated with or without 40 µM tioconazole (TC) (n = 3). C) Kaplan–Meier survival curves of MLLT3‐KMT2A fusion gene‐induced AML model mice intraperitoneally injected with 60 mg kg^−1^ tioconazole (TC) or an equivalent volume of vehicle every 3 days (n = 16 per group). D) Colony‐forming assay of mice bone marrow derived AML cells expressing ATG4B‐targeted shRNA(shATG4B) or scrambled shRNA(shNC) (n = 3). E,H,K) Kaplan–Meier survival curves of serially transplanted AML model mice with (shATG4B) or without (shNC) ATG4B deficiency at the 1st transplantation, 2nd transplantation, and 3rd transplantation (n =15 per group). F,I,L) Western blot analysis of phosphorylated CHK1 levels in AML cells derived from AML model mice with (shATG4B) or without (shNC) ATG4B deficiency at the 1st transplantation, 2nd transplantation, and 3rd transplantation. G,J,M) Cell cycle ratios of bone marrow derived NC/shATG4B AML cells from AML model mice at the 1st transplantation, 2nd transplantation, and 3rd transplantation (n = 6 per group). All the statistical data are presented as mean ± SEM. Statistical analysis was performed using two‐way ANOVA (A), two‐tailed unpaired t‐test (B, D, G, J, M), and log‐rank (Mantel–Cox) test (E, H, K). ^**^ *p*< 0.01, ^***^ *p*< 0.001.

To further investigate the function of ATG4B in leukemia progression, we inhibited ATG4B activity using an ATG4B inhibitor and gene knockdown in mouse AML cells and assessed cell proliferation. The results showed that ATG4B inhibition significantly reduced the proliferation and clonogenic potential of AML cells but had a minimal effect on autophagy (**Figure** **7**A,B,D; Figure S6A,B, Supporting Information). This suggests an autophagy‐independent role for ATG4B in the progression of MLLT3‐KMT2A‐positive leukemia. To further verify the function of ATG4B in leukemia progression in vivo, 2 × 10^5^ MLLT3‐KMT2A‐overexpressing mouse AML cells were transplanted into recipient mice (hereafter referred to as AML model mice) and treated with or without the ATG4B inhibitor tioconazole.^[^ ^17^ ^]^ The survival of AML model mice was assessed. Treatment with the ATG4B inhibitor significantly reduced the AML (GFP^+^) cell population in the peripheral blood and extended the survival of AML model mice (Figure 7C; Figure S6C, Supporting Information). Similarly, compared to WT AML model mice, ATG4B‐deficient AML model mice exhibited a markedly decreased leukemia burden and prolonged survival even after serial transplantation (Figure 7E,H,K; Figure S6D,E, Supporting Information). Meanwhile, with continuous transplantation, the lifespan of the WT AML model mice gradually decreased, indicating the malignant evolution of AML cells. Interestingly, comparable longevity of ATG4B‐deficient AML model mice was observed after serial transplantation, suggesting a lower rate of malignant evolution in ATG4B‐deficient AML cells (Figure 7E,H,K). Consistent with the lower malignant evolution, compared to WT AML cells, a significantly lower mutation burden was observed in ATG4B‐deficient AML cells after serial transplantation, as demonstrated by the lower frequencies of insertions/deletions and copy number variations in ATG4B‐deficient AML cells (Figure S6J, Supporting Information). Additionally, ATG4B deficiency in AML cells significantly promoted the activity of the S‐phase checkpoint kinases‐CHK1 (Figure 7F,I,L) and increased the number of AML cells arrested in the S phase (Figure 7G,J,M; Figure S6F–H, Supporting Information), suggesting an enhanced S‐phase checkpoint response in ATG4B‐deficient AML cells. Collectively, these findings suggest a promoting role of ATG4B in AML progression.

### 2.9 Nuclear Translocation of ATG4B Contributes to the Progression of Human AML

**Figure 8:**
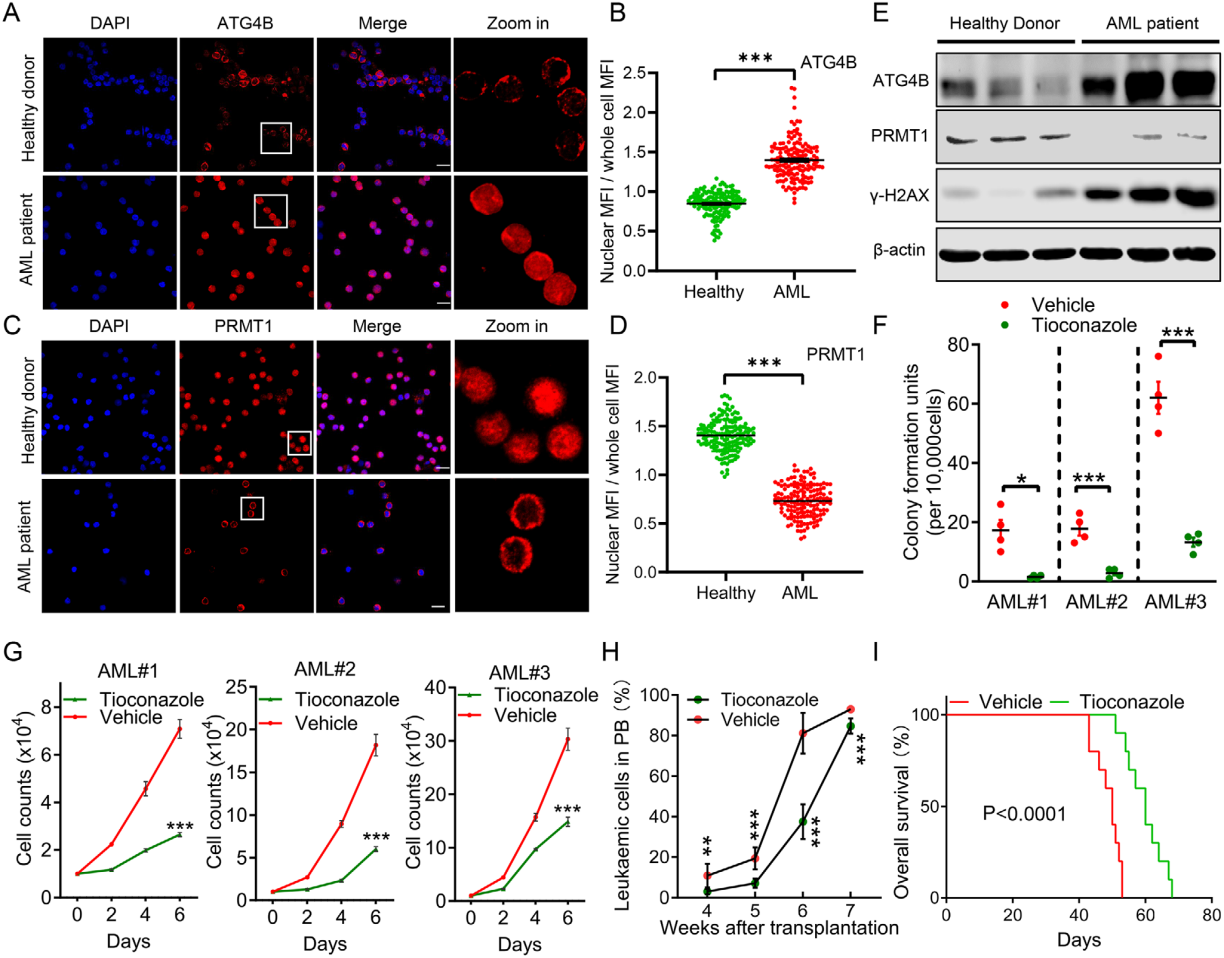
Nuclear translocation of ATG4B contributes to primary human AML cell proliferation. A,B) Representative images and quantification of ATG4B distribution in AML patient‐derived primary peripheral blood mononuclear cells (PBMCs) (n ≥ 150 cells). Scale bar, 10 µm. C,D) Representative images and quantification of PRMT1 distribution in AML patient‐derived primary PBMCs (n ≥ 150 cells). Scale bar, 10 µm. E) Immunoblot detecting ATG4B, PRMT1, and γ‐H2AX protein levels in healthy donor‐ or AML patient‐derived primary PBMCs. F) Colony‐forming assay of AML patient bone‐marrow‐derived primary CD34^+^ cells treated with or without tioconazole. G) Growth curves of AML patient bone‐marrow‐derived CD34^+^ cells treated with or without tioconazole. H) Percentages of human CD45^+^ AML cells in peripheral blood (PB) at the indicated time points after transplantation in AML patient‐derived xenografts (PDX) mice (n = 10 per group). I) Kaplan–Meier survival curves of AML‐PDX mice intraperitoneally injected with 60 mg kg^−1^ tioconazole or an equivalent volume of vehicle every 3 days (n = 10 per group). All the statistical data are presented as mean ± SEM. Statistical analyses were performed using two‐way ANOVA (G), two‐tailed unpaired t‐test (B, D, F, H), and log‐rank (Mantel–Cox) test (I). ^**^ *p*< 0.01, ^***^ *p*< 0.001.

To further confirm the role of ATG4B in the progression of human AML, we assessed the protein levels and nuclear translocation of ATG4B in primary peripheral blood mononuclear cells (PBMCs) derived from patients with AML. Compared to healthy donors, nuclear ATG4B levels were significantly higher in AML patient‐derived PBMCs, accompanied by a marked decrease in nuclear PRMT1 levels (**Figure** **8**A–D). Meanwhile, we found that ATG4B protein levels were aberrantly elevated in AML patient‐derived primary PBMCs, whereas PRMT1 levels were notably decreased and DNA damage was significantly increased (Figure 8E). Next, we investigated the role of ATG4B in human AML cell proliferation. ATG4B activity was inhibited using tioconazole in AML patient‐derived primary CD34^+^ cells and AML cell lines, and cell proliferation was assessed by colony formation and growth curve assays. Consistently, ATG4B inhibition significantly reduced colony formation and cell growth of human AML cells (Figure 8F,G; Figure S6I, Supporting Information). Lastly, we investigated the role of ATG4B in leukemia progression using AML patient‐derived xenografts (PDX). PDX mice were treated with or without the ATG4B inhibitor tioconazole, and both the AML cell burden in the peripheral blood of PDX mice and the survival of PDX mice were assessed over time. Treatment with an ATG4B inhibitor significantly reduced the leukemia burden and extended the overall survival of the PDX mice (Figure 8H,I). Taken together, these findings suggest a role for ATG4B in promoting the progression of human AML.

## 3 Discussion

Aberrant energy metabolism and DNA damage accumulation are involved in various pathophysiological processes, including aging and tumorigenesis. In the present study, we established a direct connection between energy metabolism and DNA repair in the context of energy deficiency and leukemia progression. Our findings demonstrated that during energy deficiency, ATG4B translocates from the cytoplasm to the nucleus, thereby impairing DNA repair by affecting the PRMT1‐MRE11 pathway. This defect in DNA repair mediated by ATG4B nuclear translocation is significantly increased in AML cells and contributes to AML progression.

Genomic instability plays a critical role in the development of various malignancies, including AML, particularly therapy‐related AML.^[^ ^29^, ^30^, ^31^ ^]^ Defects in DNA repair in Fanconi anemia highlight impaired DNA repair in hematopoietic stem cells, which directly exacerbate clonal hematopoiesis, myelodysplastic syndrome, and AML progression.^[^ ^32^, ^33^ ^]^ In MLL‐rearranged‐induced AML, replication‐associated DNA damage leads to DNA damage response activation and affects leukemia malignant progression.^[^ ^34^, ^35^ ^]^ In addition, various mutations in DNA damage response genes, such as *RAD51*, *XRCC1*, *XPD*, are associated with an increased risk of AML.^[^ ^32^, ^36^, ^37^ ^]^ Conversely, many AML oncogenes, such as *MLL* fusions, *FLT3‐ITD*, *N*‐*RAS*, and *TP53*, have been reported to cause DNA damage accumulation.^[^ ^4^, ^34^, ^38^, ^39^, ^40^, ^41^ ^]^ ATG4B has been implicated in the tumorigenesis of various malignancies, including osteosarcoma, breast cancer, gastric cancer, and chronic myeloid leukemia, by interfering with cell proliferation and autophagy.^[^ ^21^, ^22^, ^23^, ^24^ ^]^ However, in this study, our data showed that ATG4B was upregulated in AML cells, but that ATG4B inhibition had only a weak effect on AML cell autophagy, suggesting an autophagy‐independent role for ATG4B in AML progression. This is consistent with previous reports showing that ATG4B exhibits overlapping redundancies in autophagy in some cancer cells.^[^ ^42^, ^43^ ^]^ In this study, we provide evidence demonstrating a novel role of ATG4B, beyond its involvement in autophagy, in the progression of AML by reducing PRMT1, which plays a critical role in DNA damage repair. Inhibition of ATG4B enhances PRMT1‐MRE11‐mediated DNA damage responses, promotes S‐phase checkpoint activation, and induces robust S‐to‐G2/M cell cycle arrest in AML cells. This arrest prevents damaged cells from completing mitosis, thereby suppressing AML cell proliferation, prolonging the survival of mice with MLLT3‐KMT2A‐induced AML, and those bearing AML PDX. ATG4B inhibition shifted genomically unstable AML cells from unregulated proliferation to a stringent cell cycle check, effectively alleviating the mutational burden and malignant evolution of AML cells. These findings suggest that ATG4B may serve as a potential therapeutic target for AML.

Tumor cells are often exposed to nutrient‐ and oxygen‐poor environments,^[^ ^44^ ^]^ suggesting a potentially energy‐deficient state. Given that DNA repair requires sufficient energy, there may be a mechanistic link to the genomic instability observed in various cancers. In this study, we demonstrated a link between an aberrant energy supply, cellular DNA repair defects, and leukemia progression by promoting the translocation of ATG4B from the cytoplasm to the nucleus. This suggests a crosstalk between energy metabolism and the malignancy of tumor cells, likely through the impairment of DNA repair. AML is characterized by accumulation of DNA‐damaged immature myeloid precursors.^[^ ^45^ ^]^ Leukemia stem cells (LSCs) are a population of malignantly transformed hematopoietic stem cells. In human AML, LSCs exhibit impaired spare respiratory capacity for oxidative phosphorylation (OXPHO) compared to normal hematopoietic stem cells (HSCs).^[^ ^46^ ^]^ While HSCs can increase glycolysis to compensate for the reduced OXPHO, LSCs have minimal glycolytic reserve capacity.^[^ ^47^ ^]^ Thus, it is plausible that energy deficiency or defective energy metabolism imposes a burden of DNA damage on LSCs, driving their malignant transformation.

From an evolutionary perspective, the ability of a single protein to perform multiple functions is a valuable strategy for organisms to conserve their genetic and metabolic resources.^[^ ^48^ ^]^ Many autophagy‐related proteins play multiple roles beyond their canonical functions. ATG16 primarily facilitates the binding of LC3 to PE in autophagic vesicles to form LC3‐II by forming a complex with ATG5 and ATG12. ATG16 also plays important roles in intracellular trafficking, including exocytosis, membrane repair, and hormone secretion.^[^ ^49^, ^50^, ^51^ ^]^ UVRAG, which binds to BECN1 to stimulate autophagy, plays a role in maintaining genomic stability by interacting with the catalytic subunit of the DNA‐dependent protein kinase complex to promote DNA repair.^[^ ^52^ ^]^ In addition, UVRAG regulates centrosome function by binding to centrosomal protein.^[^ ^53^ ^]^ BECN1 has also been reported to regulate endocytic trafficking^[^ ^54^ ^]^ and EGFR signaling.^[^ ^55^ ^]^ In this study, we revealed a non‐canonical role for ATG4B beyond its involvement in autophagy. In addition to its canonical function in regulating the autophagic cycling of LC3 in the cytoplasm,^[^ ^16^, ^17^ ^]^ we found that a portion of ATG4B translocates to the nucleus and impairs the DNA repair activity of the cell under energy‐deficient conditions. While our study confirms that ATG4B undergoes nuclear translocation during energy deficiency (Figure 1), the precise molecular mechanism remains unclear. Several key observations highlight the complexity of this process: 1) Although AMPK and eIF2α are well‐established mediators of cellular stress responses,^[^ ^56^ ^]^ our data indicate that their activation is not required for ATG4B nuclear translocation. This suggests that ATG4B localization is regulated independently of these canonical energy‐sensing pathways. 2) Mass spectrometry revealed starvation‐induced changes in ATG4B post‐translational modifications (reduced Ser34 phosphorylation and increased Lys32 acetylation). However, mutagenesis studies (S34A/S34D, K32R) demonstrated that these modifications alone do not govern ATG4B nuclear import (data not shown), implying the involvement of alternative regulatory mechanisms. 3) Bioinformatics analysis did not identify classical nuclear localization signals or nuclear export signals within ATG4B, further supporting the hypothesis that its translocation may occur via non‐canonical mechanisms. Given these findings, we propose that ATG4B nuclear translocation may depend on its interaction with other nucleocytoplasmic shuttling proteins, which could facilitate ATG4B nuclear entry. Future studies should employ proteomic approaches to identify ATG4B‐interacting proteins that are required for ATG4B nuclear translocation during energy deficiency. DNA repair is a highly energy‐demanding process.^[^ ^11^, ^12^, ^13^ ^]^ Starvation results in a significant energy shortage in cells. One likely physiological effect of ATG4B nuclear translocation‐mediated inhibition of DNA repair is that cells downregulate DNA damage repair activity to reduce energy expenditure. This adaptation helps maintain the basic requirements for cell survival during starvation.

In summary, this study revealed a novel oncogenic role of ATG4B in the cell nucleus, where it inhibits DNA repair and plays an important role in the progression of malignancy. Hence, inhibition of ATG4B nuclear localization could provide new opportunities for cancer prevention and therapy. Finally, our study underscores the need for further exploration of the various roles of ATG4B to fully understand its implications in cancer biology and its potential as a therapeutic target.

## 4 Experimental Section

### Reagents, Antibodies, and Plasmids

Torin1 (475911) and Compound C (P5499) were purchased from Sigma.G418 (11811031) were purchased from ThermoFisher. ATP (S5260), Etoposide (S1225), and 2 ‐ DG (S4701) were purchased from Selleck. Tioconazole (B2051) was purchased from APExBIO. Puromycin (A610593 ‐ 0025) was purchased from Sangon Bio. The following antibodies were used: anti‐GFP (Santa Cruz, sc9996), anti‐LC3 (Sigma, L7643), anti‐ATG4B (Sigma, A2981), anti‐γ‐H2AX, (Cell signaling Technology, 9718), anti‐53BP1 (Novus, NB100‐304), anti‐MRE11 (Novus, NB110‐142), anti‐PRMT1 (Cell signaling Technology, 2449) anti‐ADMA (Cell signaling Technology, 13522), anti‐β‐actin (Sigma, A5316), anti‐laminB1 (Abcam, ab133741), anti‐α‐tubulin (Abcam, ab52866), anti‐histone H3.1 (Bioss, bs‐17422R), anti‐Phospho‐eIF2α (Cell signaling Technology, 3398).

For plasmid construction, mCherry‐LC3B and GFP‐ATG4B have been described previously.^[^ ^17^ ^]^ To generate NLS‐ATG4B and NES‐ATG4B constructs, oligonucleotides encoding three tandem repeats of nuclear localization signal (NLS: GATCCAAAAAAGAAGAGAAAGGTAGATCCAAAAAAGAAGAGAAAGGTAGATCCAAAAAAGAAGAGAAAGGTA) and nuclear export signal (NES: CTTGCAC‐TCAAGGCGGGCTTGGATATCCTTGCACTCAAGGCGGGCTTGGATATCCTTGCACTCAAGGCGGGCTTGGATATC) were synthesized, annealed, and inserted into GFP‐ATG4B. For PRMT1 expression constructs, cDNAs of PRMT1 were subcloned by PCR and subsequently transferred to pLV‐Hygro vectors. The ATG4B ShRNA targeted to the sequence 5’‐CCATCCATCAGATAGCGCAAA‐3’, was subcloned to SF‐LV‐ShRNA‐mCherry vector. All constructs were validated by sequencing.

### Cell Culture, Transfection, and Starvation

HEK293 (RRID: CVCL_0045) and HEK293T (RRID: CVCL_0063) cells were cultured in Dulbecco's Modified Eagle Medium (DMEM, Gibco, C11995500BT), while THP‐1 (RRID: CVCL_0006) and KG‐1 (RRID: CVCL_0374) cells were maintained in RPMI‐1640 medium (Gibco, 11875093), both supplemented with 10% fetal bovine serum (FBS, Excell Bio) under standard culture conditions (37 °C, 5% CO_2_). HEK293 and HEK293T cells were described previously.^[^ ^57^ ^]^ THP‐1 and KG‐1 cells were newly purchased from Ubigene Biosciences Co., Ltd. All cell lines were authenticated by STR profiling and were cultured free from mycoplasma contamination. Primary MLLT3‐KMT2A^+^ AML cells were cultured in StemSpan Serum‐Free Expansion Medium (SFEM) (StemCell Technologies, 09650), supplemented with low concentrations of growth factors (GFs) similar to those present in long‐term BM culture stroma‐conditioned medium (50 ng mL^−1^ Mouse Recombinant stem cell factor [mSCF] (PeproTech, 250‐03), 10 ng mL^−1^ Mouse Recombinant thrombopoietin [mTPO] (PeproTech, AF‐315‐14), 10 ng mL^−1^ Mouse Recombinant interleukin 3 [mIL‐3] (PeproTech, 213‐13), and 10 ng mL^−1^ Mouse Recombinant interleukin‐6 [mIL‐6] (PeproTech, 216‐16)). Primary AML patient bone marrow‐derived CD34+ cells were cultured in SFEM supplemented with Human Recombinant interleukin 3 [rhIL3] (20 ng mL^−1^) (PeproTech, 200‐03), FLT3 ligand [rhFLT3L] (100 ng mL^−1^) (PeproTech, 300‐19), thrombopoietin [rhTPO] (50 ng mL^−1^) (PeproTech, AF‐300‐18‐10), stem cell factor [rhSCF] (100 ng mL^−1^) (PeproTech, 300‐07). Transient transfection was performed using Lipofectamine 3000/2000 according to the manufacturer's instructions. Cells stably expressing mCherry‐LC3B were created by transient transfection followed by selection with G418 (500 µg mL^−1^) (Gibco, 11811031). Cells coexpressing LC3B with GFP, GFP‐WT‐ATG4B, GFP‐NES‐ATG4B, or GFP‐NLS‐ATG4B were created by transfecting the protein into the LC3B stable cell line, and were sorted by the FACS Aria III cell sorter (BD Biosciences) according to the GFP fluorescence or selected with puromycin (1 µg mL^−1^) (BBI, A610593‐0025). Triple‐expressing cell lines (Flag‐PRMT1 with LC3B and GFP‐ATG4B variants) were established by transfecting Flag‐PRMT1 into GFP‐ATG4B/LC3B dual‐stable lines, followed by hygromycin B selection (200 µg mL^−1^) (Thermo Fisher, 10687010) for at least 14 days, with validation via western blotting. For 2‐DG treatment, cells were treated with 2mm 2‐DG in DMEM medium supplemented with 10% FBS for 24 h. For cell starvation treatment, cells were washed twice with pre‐warmed (37 °C) phosphate‐buffered saline (PBS) and then incubated in starvation medium (140 mm NaCl, 1 mm CaCl_2_, 1 mm MgCl_2_, 5 mm Glucose, 20 mm Hepes, 1% BSA, pH 7.4).

### Lentivirus Production and Infection

Lentivirus was produced in 293T cells after transfection of 15 mg SF‐LV‐ShRNA‐mCherry plasmid, 12 mg PSPAX2 plasmid, and 4 mg PMD2G plasmid. The virus was collected 48 h after transfection and concentrated by centrifugation at 25 000 rpm for 2.5 h at 4 °C in SW‐32Ti rotor, and the virus pellet was suspended in PBS. MLLT3‐KMT2A^+^ AML cells from primary AML mice were cultured in SFEM supplemented with growth factors including mSCF, mTPO, mIL‐3, and mIL‐6. Lentivirus suspensions were added into cells according to titration results, followed by isolation of GFP/mCherry double‐positive populations 48 h post‐infection using a FACS Aria III sorter.

### Patient Samples

The AML patient and healthy donor samples were collected from the surplus peripheral blood and bone marrow aspirations at the time of diagnosis with informed consent and supplied by the Department of Hematology at the First Affiliated Hospital of Jinan University, Guangdong Second Provincial General Hospital, or the Sixth Affiliated Hospital, Sun Yat‐sen University. (AML patients' diagnosis information in Table S1, Supporting Information). The experiments involving human samples were conducted according to the relevant ethical regulations and approved by the Medical Ethics Committees of Jinan University (Approval No. JNUKY‐2024‐0061).

### Immunoprecipitation and Western Blot

For immunoprecipitation, cells were lysed in NP‐40 buffer (50 mm Tris‐HCl pH 7.4, 150 mm NaCl, 1% NP‐40, 1 mm EDTA) containing protease inhibitors; GFP‐tagged proteins were immunoprecipitated using GFP‐Trap nano beads (ChromoTek, gta‐100), while endogenous proteins were captured by antibody with Protein A/G beads (Thermo Fisher, 88802) through overnight incubation at 4 °C. Immunocomplexes were washed and analyzed by Western blot. For Western blot, the proteins from lysed cells were denatured and separated with SDS‐PAGE. Then, the proteins were transferred to nitrocellulose membranes, blocked, and incubated with the corresponding primary antibodies and species matched LI‐COR fluorescent secondary antibodies. The specific bands were analyzed by Western blot imaging system (LI‐COR Biosciences).

### Immunostaining and Confocal Microscopy

After treatment, cells were immediately fixed for 10 min with 4% Paraformaldehyde (Sigma, P6148) at room temperature, followed by three washes with 1× PBS. Subsequently, the cells were blocked for 30 min at room temperature in PBS containing 10% FBS and 0.5% Triton X‐100 (Sigma, 93443). Primary antibodies were incubated overnight at 4 °C and then washed three times with 1× PBS. The corresponding secondary antibodies were incubated for 2 h at 37 °C and then washed three times with 1× PBS. For suspension cells, the cells were stained as above in a 1.5 mL centrifuge tube, and finally, the cells were centrifuged onto slides for observation. Images were captured with a Zeiss LSM880 laser scanning confocal microscope (Carl Zeiss) with a 63× Plan Apochromat 1.4 NA objective. Nuclear foci of γ‐H2AX and 53BP1 in HEK293 cells were quantified using VolocityDemo software, while fluorescence intensity analysis was performed using ZEN 2.0 (Carl Zeiss): nuclear boundaries were defined by DAPI, whole‐cell areas demarcated by cytoplasmic signals, and nuclear‐to‐whole‐cell mean fluorescence intensity (MFI) ratios calculated from ≥ 50 cells per sample to evaluate nuclear translocation. For live‐cell imaging, cells in glass‐bottom dishes (NEST, 801001) with starvation medium (140 mm NaCl, 1 mm CaCl_2_, 1 mm MgCl_2_, 5 mm glucose, 20 mm HEPES, 1% BSA, pH 7.4) were maintained at 37 °C/5% CO_2_ using a Zeiss incubation chamber mounted on an LSM880 confocal microscope. Time‐lapse imaging was performed at 1‐min intervals using a 63× Plan‐Apochromat oil objective (NA 1.4) with 488 nm laser excitation, while fluorescence recovery after photobleaching (FRAP) assays were conducted by completely bleaching GFP signals in defined regions using high‐intensity 488 nm laser (100% power, 10–30 iterations), followed by time‐lapse acquisition at specified intervals to monitor signal recovery.

### Comet Assay

Cells were treated with DNA‐damaging agents, harvested, and the comet procedure was applied using the Comet Assay experimental system (Trevigen, 4250‐050‐K). For that, cells were mixed with low‐melting agarose, and the cell suspension was overlaid on microscope slides. Cell lysis was carried out within the agarose. After lysis, electrophoresis of the DNA trapped in the agarose was performed at 1 V cm^−1^ for 45 min. After staining the slides with SYBR Green dye for 10 min, images of 50 randomly selected cells per sample were captured using a Zeiss LSM510 Meta (Carl Zeiss) with a 10× objective. The relative length and intensity of DNA tails relative to heads were proportional to the amount of DNA damage in individual nuclei. These parameters were measured observer‐independent and in an unbiased fashion by tail moment quantification with TriTek Comet Score software (TriTek Corp., Sumerduck, VA). Representative images of single cells from comet slide samples were obtained post‐analysis.

### ATP Detection

Intracellular ATP content of bone marrow derived MLLT3‐KMT2A^+^ AML cells was performed according to the manufacturer's instructions (Abcam, ab113849). Briefly, 1 × 10^5^ bone marrow derived c‐kit^+^ MLLT3‐KMT2A^+^ AML or normal cells were lysed with detergent buffer for 10 min at room temperature, then mixed with substrate solution for 5 min, followed by dark incubation for 10 min and measurement of luminescence.

### AML Model Mouse

For the generation of MLLT3‐KMT2A‐driven AML model mouse, c‐Kit^+^‐hematopoietic stem/progenitor cells (HSPCs) were enriched from wild‐type C57BL/6 mouse bone marrow using an APC‐conjugated c‐Kit antibody (Invitrogen, 17‐1171‐83) and anti‐APC MicroBeads (Miltenyi Biotec, 130‐090‐855), and then cultured in IMDM (Gibco, 12440053) containing 10% FBS, 50 ng mL^−1^ mSCF, 10 ng mL^−1^ mIL‐3, and 10 ng mL^−1^ mIL‐6 overnight to stimulate cell proliferation. The next day, cells were infected with replication‐incompetent retroviral vectors encoding MLLT3‐KMT2A fusion protein (GFP^+^). After 48 h, cells were harvested and transplanted into lethally irradiated recipient mice. The primary AML mice were monitored for MLLT3‐KMT2A AML development. For the AML‐patient‐derived xenograft (PDX) model mouse, 1×10^7^ AML patient‐derived cells were injected into the orbital vein of irradiated (1Gy) B‐NDG mice. The primary AML‐PDXs mice were monitored for AML development.

### Survival Curve

For the AML mouse model to Tioconazole treatment, 2 × 10^5^ MLLT3‐KMT2A^+^ AML cells from primary AML Mice were injected into the orbital vein of WT C57BL/6J mice. One week after transplantation, mice were treated with Tioconazole (60 mg kg^−1^/3day, i.p) or equivalent vehicle. Then the survival time of the mice was recorded. For the WT or ATG4B deficiency AML mouse model, 4 × 10^5^ MLLT3‐KMT2A^+^ AML cells with or without ATG4B knockdown were injected into the orbital vein of irradiated (4.5Gy) WT C57BL/6J mice. Then the survival time of the mice was recorded. For AML‐PDXs model to Tioconazole treatment, 1 × 10^7^ AML cells from primary AML‐PDXs mice were injected into the orbital vein of irradiated (1Gy) B‐NDG mice. One week after transplantation, mice were treated with Tioconazole (60 mg kg^−1^/3day, i.p) or equivalent vehicle. Peripheral blood chimerism rate of the AML cells was analyzed 2 or 4 weeks later by flow cytometry (LSRFortessa; BD). All mice were housed in a specific pathogen‐free environment in the Laboratory Animal Center at Jinan University. The Animal Care and Ethics Committee of Jinan University approved all animal experiments in this study (Approval No. IACUC‐20221116‐07).

### Colony‐Forming Assay

Mice bone marrow derived MLLT3‐KMT2A^+^ AML cells were plated in methylcellulose medium (M3534, StemCell Technologies, 03534) according to the manufacturer's instructions, supplemented with 100 IU mL^−1^ penicillin and 100 mg mL^−1^ streptomycin. Colonies were evaluated after seven days of incubation. Primary AML patient bone marrow derived CD34+ cells were plated in methylcellulose medium (MethoCult H4434; StemCell Technologies, 04434) according to the manufacturer's instructions, and colonies were evaluated after 10 days of incubation.

### Cell Cycle and Cell Count Assay

For the cell cycle assay, mouse bone marrow derived MLLT3‐KMT2A^+^ AML cells with or without ATG4B knockdown were fixed by 75% ethanol at 4 °C overnight. Then the cells were stained with DAPI (5 µg mL^−1^, Sigma, D9542) in the dark for 30 min at room temperature, and the cell cycle was measured by BD LSRFortessaTM cell analyzer (BD Biosciences). The ModFit LT software (version 5) was used to obtain the information of the cell cycle. For cell count assays, mouse bone marrow‐derived MLLT3‐KMT2A^+^ AML cells, primary AML patient‐derived CD34^+^ cells, or THP‐1/KG‐1 cell lines were seeded in 24‐well plates at cell type‐specific densities (1 × 10⁴–5 × 10⁴ cells per well) with five technical replicates per experimental group in 0.5 mL complete medium containing Tioconazole (20–80 µM) or vehicle control (DMSO, Sigma, D2650), with three independent plates designated for termination at 24, 48, and 72‐h timepoints respectively; at each interval, cells were suspended in 0.04% trypan blue (Gibco, 15250061), and viable cells (viability > 90% by exclusion criteria) quantified using a blood cell counting plate.

### Analysis of Public Databases

For TCGA‐AML RNA‐seq data and GTEx normal control data, the GEPIA2 interactive platform (https://gepia2.cancer‐pku.cn) was utilized to achieve processed results. Additional datasets, including BloodSpot dataset(https://www.fobinf.com/), Beat AML (https://registry.opendata.aws/beataml.), and GEO datasets (GSE74912, GSE12417, GSE83533), were directly downloaded as processed RNA‐seq matrices with matched clinical annotations from their respective repositories. Survival curves were analyzed using the Log‐rank (Mantel‐Cox) test, correlations were assessed via Pearson's method, and group comparisons were performed with two‐tailed unpaired *t*‐tests. All statistical analyses and visualizations were conducted using GraphPad Prism (v9.0).

### Statistical Analyses

All statistical analyses were conducted using GraphPad Prism 9.0 software (GraphPad), with experimental design‐specific statistical tests (parameters and number of replicates are indicated for each experiment in the figure legends). For pairwise comparisons between independent groups, variance homogeneity was first assessed via F‐test, with standard two‐tailed unpaired Student's t‐tests applied when equal variance assumptions were satisfied, and Welch's corrected two‐tailed t‐tests employed when unequal variances were detected. Two‐way ANOVA with Tukey's post hoc test was employed for multifactorial comparisons to assess interactions between two independent variables. Log‐rank tests analyzed statistical differences in Kaplan–Meier survival curves. Pearson correlation coefficients (r‐values) quantified linear relationships between continuous variables, with significance thresholds reflecting covariance strength. For all analyses, data are presented as mean ± SEM. Statistical significance thresholds were defined as follows: ^*^ *p* < 0.05, ^**^ *p* < 0.01, ^***^ *p* < 0.001, and non‐significant (ns) designated for p ≥ 0.05. Additional methods, such as flow cytometry analysis, in vitro proteolysis assay, and others, used in this article are provided in Supporting Methods.

## Conflict of Interest

The authors declare no conflict of interest.

## Author Contributions

B.L. and Z.K.W. designed the study. Z.K.W., B.L., X.L.Z., and Y.Y.Z. Performed experiments and analyzed data. B.L., Z.K.W., and Z.Y.J. wrote the paper. Y.H.X., Z.Y., S.L., K.X.H., S.Q.D., J.D., and H.Z. provided the AML samples and clinical information. S.Y.L., Y.T.F., H.P.L., Q.D.G., J.W., J.P.Z., Q.H.Z., T.F.C., and X.L.G. provided additional expertise. B.L. and Z.Y.J. supervised the study.

## Supporting information



Supporting Information

Supporting Information

## Data Availability

The data that support the findings of this study are available from the corresponding author upon reasonable request.
