# Supplementary material for: Energy Deficiency‐Induced ATG4B Nuclear Translocation Inhibits PRMT1‐Mediated DNA Repair and Promotes Leukemia Progression
Source: Adv Sci (Weinh). 2025 Aug 11;12(40):e09838. doi: 10.1002/advs.202509838 (PMC12561473; doi:10.1002/advs.202509838)
Supplement: Supplementary file 1 — Supporting Information [file ADVS-12-e09838-s001.docx]

**Supplemental Methods**

**Generation of ATG4B knockout cell line**

pEP-*ATG4B*-KO plasmid was made by cloning the target DNA sequence of human *ATG4B* (CTCTGACCTACGACACTCTC) into a pEP-KO-Z1779 vector using SapI restriction sites. ATG4B knockout HEK293 cells were generated by transfection of pEP-*ATG4B*-KO plasmid followed by selection with puromycin (1 μg/mL). Single-cell clones were isolated by limiting dilution in 96-well plates and expanded for validation. Genomic DNA was extracted from candidate clones, and the target regions were amplified by PCR and sequenced to confirm frameshift mutations. Successful ATG4B ablation was further verified by Western blot using anti-ATG4B antibody (Sigma, A2981).

***In vitro* proteolysis assay**

The LC3B-mCherry substrate was purified through expression of the LC3B-mCherry plasmid in ATG4B knockout HEK293 cells (to block endogenous LC3B C-terminal processing). Cells were lysed 48 hours post-transfection using NP-40 buffer (lacking protease inhibitors) and subjected to affinity purification with mCherry Nano Beads (AlpalifeBio, KTSM1331) via 2-hour incubation at 4°C. Beads were pelleted by centrifugation and rigorously washed six times (as immunoprecipitation section described) to isolate resin-bound LC3B-mCherry. For *E.coli* protein purification, pCold1-His-ATG4B or pGEX4T1-GST-PRMT1 plasmids were transformed into BL21 competent cells. Single colonies were expanded in 500 mL cultures until OD600 reached 0.4–0.5, followed by induction with 0.1 mM IPTG (His-ATG4B: 25°C for 6 hours; GST-PRMT1: 16°C for 12 hours). Bacterial pellets were resuspended in ice-cold lysis buffer (50 mM PBS, 500 mM NaCl, 10 mM imidazole pH 7.4, supplemented with 1 mM AEBSF for His-ATG4B or 1 mM PMSF for GST-PRMT1), lysed by sonication, and clarified supernatants were incubated with HisPur Cobalt Resin (Thermo, 89964) or Glutathione Sepharose 4B (Cytiva, 17075601) for 1 hour at 4°C. Proteins were eluted using imidazole-containing buffer (50 mM PBS, 500 mM NaCl, 150 mM imidazole pH 7.4) for His-ATG4B or glutathione elution buffer (10 mM reduced glutathione in 50 mM Tris-HCl, pH 8.0) for GST-PRMT1, concentrated using 50 kDa Millipore centrifugal filters (UFC5050), and validated by SDS-PAGE with Coomassie staining alongside quantification via BCA Protein Assay Kit (Thermo, 23227). Proteolytic reactions were performed by mixing purified proteins (or resin-bound LC3B-mCherry) in reaction buffer (50 mM Tris-HCl pH 8.0, 150 mM NaCl, 5 mM DTT) at 37°C for 30 minutes. Reactions were terminated by boiling in SDS buffer for 5 minutes and analyzed by western blot as described.

**Mass Spectrometry**

For liquid chromatography-mass spectrometry (LC-MS/MS) analysis of Flag-ATG4B-associated protein complexes, HEK293T cells overexpressing Flag -ATG4B were lysed in NP-40 buffer (50 mM Tris-HCl pH 7.4, 150 mM NaCl, 1% NP-40, 1 mM EDTA) supplemented with protease/phosphatase inhibitors, and pre-cleared lysates were incubated with Flag-tagged beads (Sigma, A2220) for 2 h at 4°C with rotation; after four washes with ice-cold wash buffer (50 mM Tris-HCl pH 7.4, 150 mM NaCl, 0.1% NP-40, 1 mM EDTA) and twice wash with ice-cold PBS, immunocomplexes were eluted by boiling in 2× SDS buffer and boiled for 5 min, resolved on a 4–20% gradient SDS-PAGE gel (Genscript, M00657) until complete separation of the entire lane (tracked by protein ladder), and the entire lane was excised, and submitted to Shanghai Applied Protein Technology Co., Ltd. (China) for in-gel trypsin digestion and LC-MS/MS analysis.

**Flow Cytometry Analysis**

Bone marrow cells were isolated from femurs, tibias, and vertebral bones of AML-bearing mice through mechanical dissociation in 2% FBS/PBS. Tissue fragments were filtered through a 70-μm cell strainer, followed by red blood cell lysis using BD Pharm Lyse™ Buffer (BD Biosciences, 555899) for 5 min at room temperature. Washed cells were resuspended in 50 μL of 2% FBS/PBS and stained with fluorophore-conjugated antibodies (clone details in “Reagents, antibodies and plasmids”) for 30 min at 4°C in light-protected conditions. For peripheral blood analysis, GFP/mCherry fluorescence was directly quantified post-lysis without antibody staining. All samples were acquired on a BD LSR Fortessa™ cytometer equipped with 405 nm, 488 nm, 561 nm, and 640 nm lasers, utilizing FACSDiva™ software (BD Biosciences). data analysis and process were performed with FlowJo™ v10.8.1. The following antibodies were used: anti-human CD45-APC (Biolegend, 368512), anti-human APC-CD34 (Biolegend, 343509), anti-mouse CD4-Biotin (Biolegend, 100404), anti-mouse CD8a-Biotin (Biolegend, 100704), anti-mouse B220-Biotin (Biolegend, 103204), anti-mouse Gr-1-Biotin (Biolegend, 108404), anti-mouse TER-119-Biotin (Biolegend, 116204), anti-mouse CD11b-Biotin (Biolegend, 101203), anti-Streptavidin-APC/Cyanine7 (Biolegend, 405208), anti-mouse CD117 (c-Kit) (Invitrogen, 17-1171-83), anti-mouse Ly-6A/E (Sca-1)-PE/Cyanine7 (Biolegend, 122514), anti-mouse CD34-PerCP/Cyanine5.5 (Biolegend, 119328), anti-mouse CD16/CD32- Alexa Fluor 700 (Invitrogen, 56-0161-82), anti-mouse Ly-6G/Ly-6C (Gr-1)-PE/Cyanine7 (Biolegend, 108416), anti-mouse/human CD11b-APC (Biolegend, 101212).

**Genome-wide Sequencing Analysis**

Bone marrow cells from NC and shATG4B AML mice (n=3 per group) were pooled in equicellular ratios (5×10⁶ cells per pool), pelleted by centrifugation (300 ×g, 5 min), flash-frozen in liquid nitrogen, and stored at −80°C prior to whole-genome sequencing (WGS) conducted by Tsingke Biotech Co., Ltd (Beijing, China). Sequencing libraries were prepared using the TrueLib DNA Library Rapid Prep Kit for Illumina (ExCell Bio, NGS00-1083) with 350 bp insert sizes and sequenced on the DNBSEQ-T7 platform (10× coverage). Raw sequencing data underwent quality control (FastQC v0.11.9), alignment to mm10 reference genome, and variant calling via GATK v4.2.6.1 (Ensembl Variant Effect Predictor (VEP) for InDels; Sentieon for copy number variations). Somatic mutations with quantification of InDel and CNV counts derived from raw sequencing outputs after filtered. Mutation burdens were visualized as non-normalized heatmaps in GraphPad Prism 9.0, with chromosomal coordinates annotated for genomic localization.

**Supplemental Tables**

Supplemental Table S1 Clinical information relevant to AML samples.

| ID | FAB | Type | Risk | Status | Gene mutation | Cytogenetics | FLT3 | Used in Figure |
| --- | --- | --- | --- | --- | --- | --- | --- | --- |
| 1 | M1 | PB | A | R | IDH1, NPM1 | NK | ITD | 8A-E |
| 2 | M4 | PB | F | U | TET2,NPM1 | NK | ITD | 8A-E |
| 3 | M4 | PB | I | U | ASXL1, CEBPA, WT1 | del(9)(q13q22) | WT | 8A-E |
| 4 | M2 | BM | A | U | WT1, GATA2, CEBPA | t(11q23) | WT | 8F-G |
| 5 | M2 | BM | A | U | DNMT3A, TET2, KIT | ND | WT | 8F-G |
| 6 | M5 | BM | A | U | DNMT1, IDH1 | NK | ITD | 8F-G |
| 7 | M4 | BM | I | U | DNMT3A, NPM1, RAD21 | NK | ITD | 8H-I |
| 8 | M4 | BM | A | R | IDH2, TET2 | NK | ITD | 8H-I |

Abbreviations: BM, bone marrow; PB, peripheral blood; F, favorable-risk; I, intermediate-risk; A, adverse-risk; U, untreated; R, relapsed; ND, not determined; NK, normal karyotype; WT, FLT3-wild type; ITD, FLT3-ITD;
