# Supplementary material for: Energy Deficiency‐Induced ATG4B Nuclear Translocation Inhibits PRMT1‐Mediated DNA Repair and Promotes Leukemia Progression
Source: Adv Sci (Weinh). 2025 Aug 11;12(40):e09838. doi: 10.1002/advs.202509838 (PMC12561473; doi:10.1002/advs.202509838)
Supplement: Supplementary file 2 — Supporting Information [file ADVS-12-e09838-s002.pdf]

Supplementary Figures

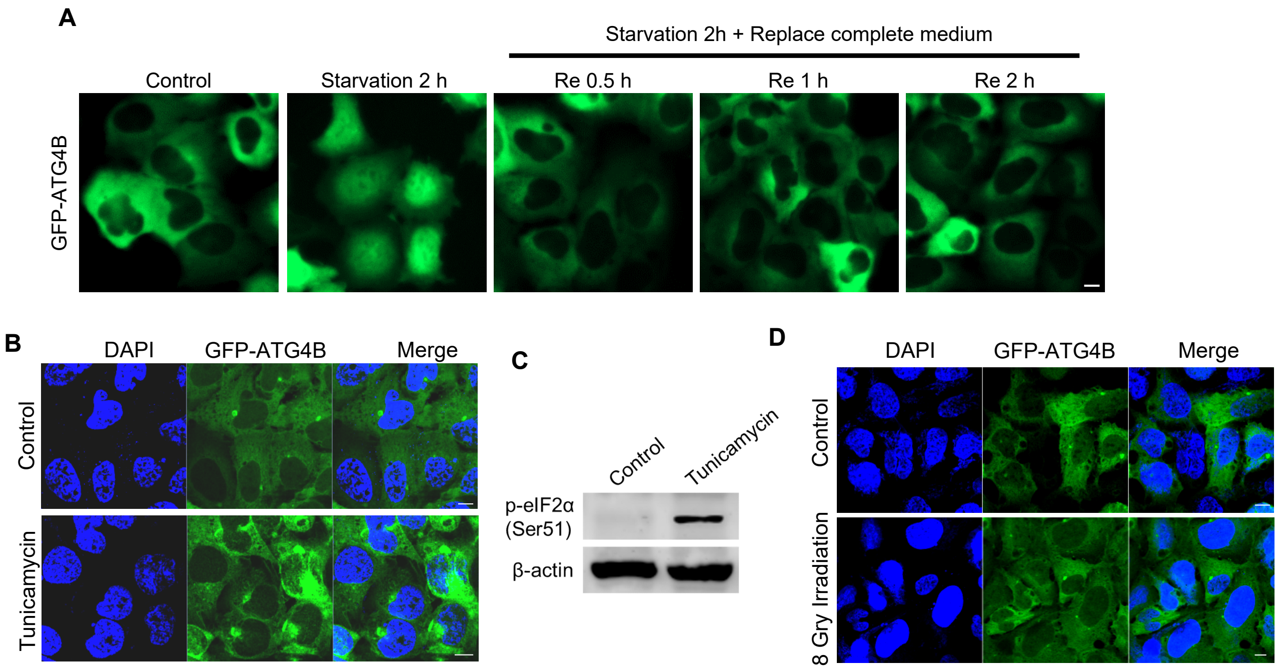

Supplementary Data Fig 1. GFP-ATG4B distribution in cells under synthesis inhibition or irradiation.

A. Representative images showing GFP-ATG4B distribution in cells subjected to starvation for 2 hours, followed by incubation in complete medium for the indicated times. Scale bar, 10μm.

B. Representative images showing GFP-ATG4B distribution in cells treated with or without 3 μg/mL Tunicamycin for 24 hours. Scale bar, 10μm.

C. Western blot detecting phosphorylated eIF2α levels in cells treated with or without 3 μg/mLTunicamycin for 24 hours.

D. Representative images showing GFP-ATG4B distribution in cells treated with 8Gy irradiation. Scale bar, 10μm.

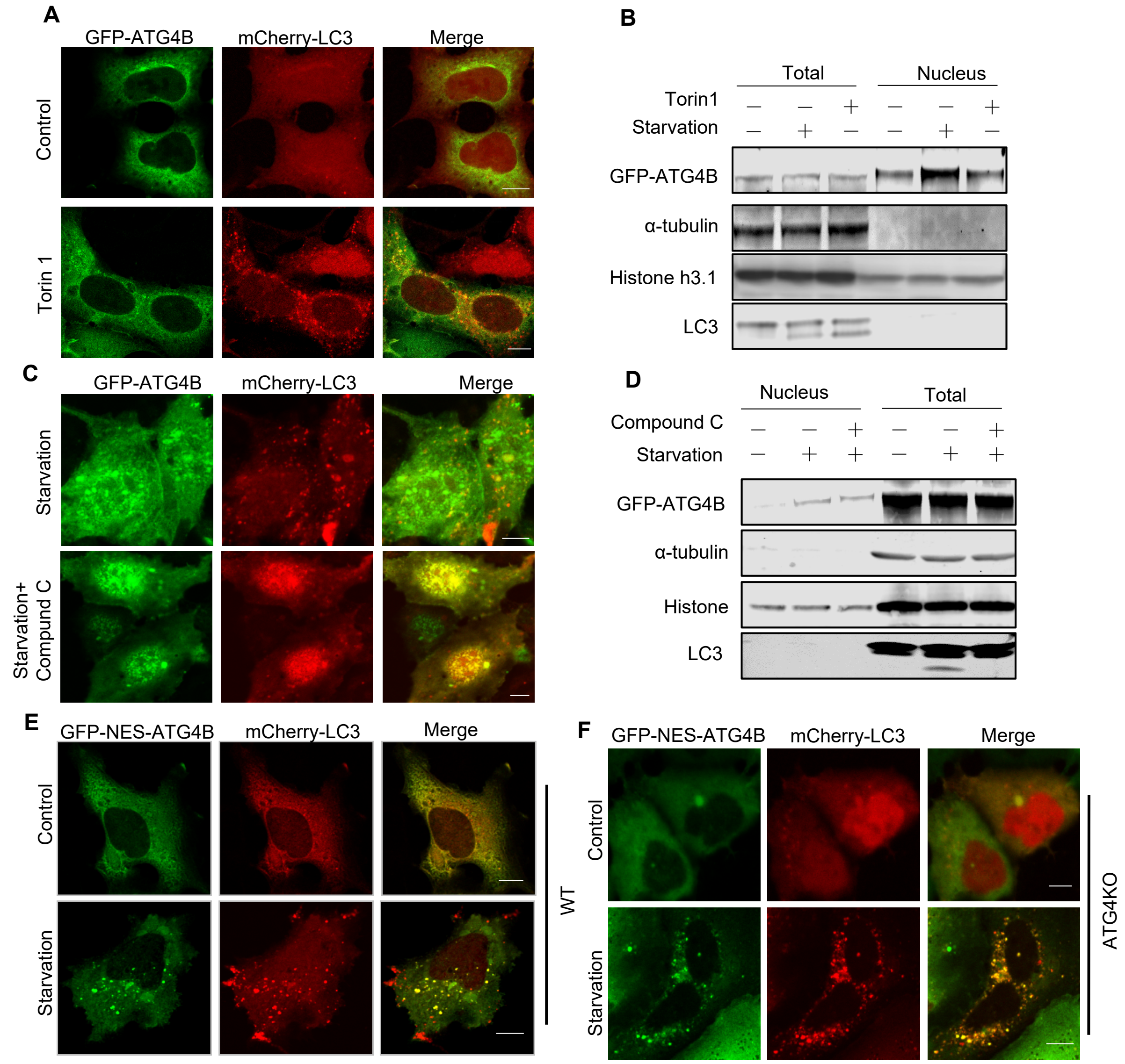

Supplementary Data Fig 2. The nuclear translocation of ATG4B is dispensable for autophagy.

A. Representative images showing GFP-ATG4B and mCherry-LC3B distribution in cells coexpressing GFP-ATG4B and mCherry-LC3B, treated with or without 250nM Torin1 for 4 hours. Scale bar, 10μm.

B. Western blot detecting nuclear ATG4B levels in cells treated with or without starvation for 2 hours, or 250nM Torin1 for 4 hours.

C. Representative images showing GFP-ATG4B and mCherry-LC3B distribution in cells coexpressing GFP-ATG4B and mCherry-LC3B, treated with starvation or starvation supplemented with 20 μM compound C treatment for 2 hours.

D. Western blot detecting nuclear ATG4B levels in cells treated with starvation or starvation supplemented with 20 μM compound C for 2 hours.

E and **F.** Representative images showing GFP-NES-ATG4B and mCherry-LC3B distribution in WT (**E**) or ATG4B KO (**F**) HEK293 cells coexpressing GFP-NES-ATG4B and mCherry-LC3B under fed condition (Control) or starvation (Starvation). Scale bar, 10μm.

A

ATG4B purification

control			starvation-total			starvation-nucleus		
Protein	# unique peptides	Coverage (%)	Protein	# unique peptides	Coverage (%)	Protein	# unique peptides	Coverage (%)
PRMT1	-	-	PRMT1	7	27	PRMT1	8	20
XRCC5	8	13	XRCC5	9	14	XRCC5	2	2
LIG3	9	9	LIG3	8	8	LIG3	1	2
PARP1	1	2	PARP1	2	3	PARP1	6	8
XRCC1	-	-	XRCC1	1	1	XRCC1	-	-
PRKDC	1	1	PRKDC	2	1	PRKDC	1	1
RAD50	-	-	RAD50	-	-	RAD50	1	1
RAD51	2	5	RAD51	1	3	RAD51	2	9
MDC1	1	0	MDC1	2	1	MDC1	1	0

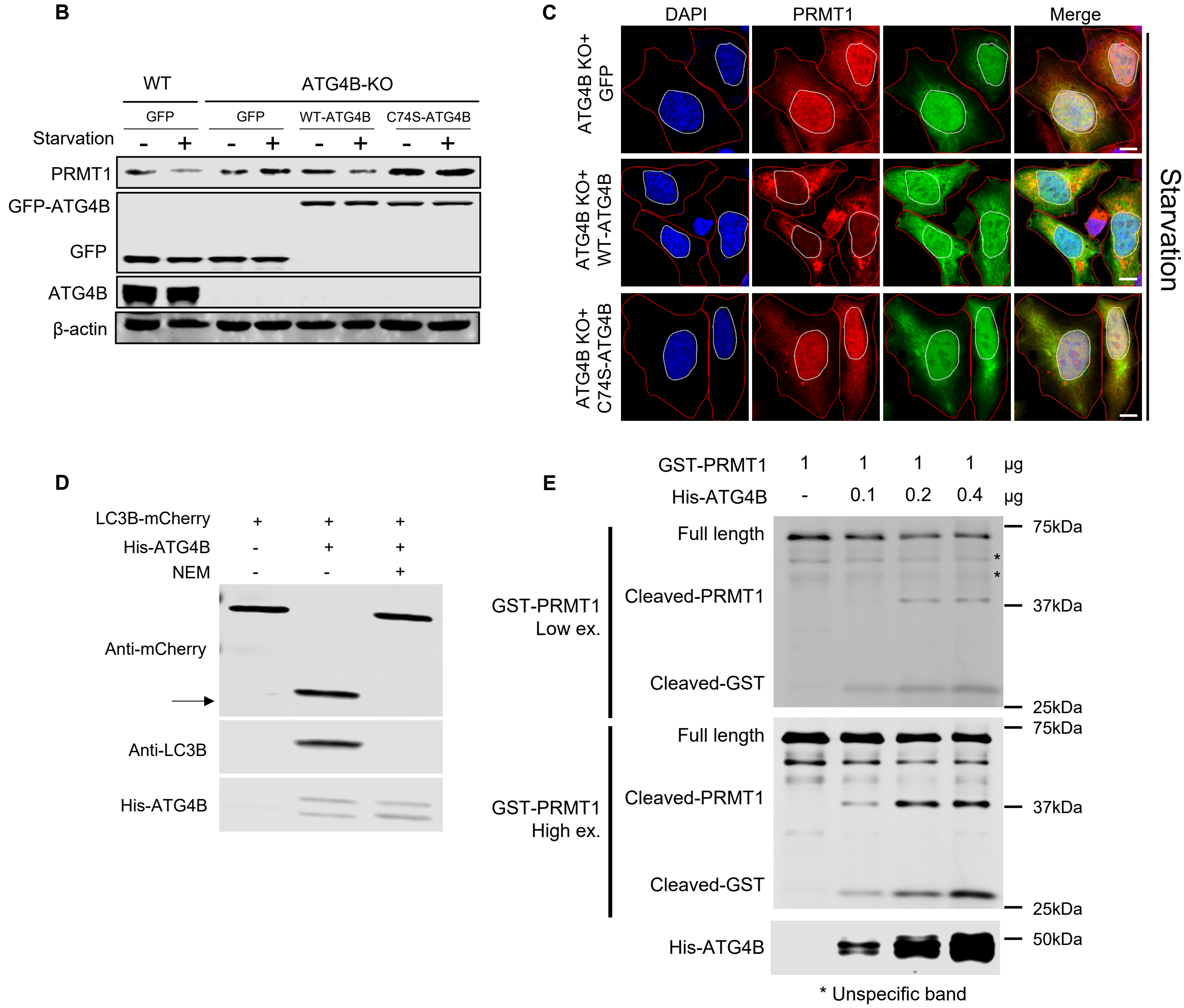

Supplementary Data Fig 3. The nuclear ATG4B interacts with PRMT1.

A. Flag-ATG4B protein was immunoprecipitated from HEK293T cells expressing with Flag-ATG4B. Co-precipitated proteins were analyzed by mass spectrometry. The graphs shows proteins associated with DNA repair identified by mass spectrometry as ATG4B binding partners, along with their unique peptides and coverage.

B. Western blot detecting PRMT1 levels in wild type or ATG4B KO cells expressing GFP, WT-ATG4B or C74S-ATG4B respectively, under starvation for 4 hours or without treatment.

C. Representative images showing nuclear PRMT1 levels and GFP-ATG4B distribution under starvation in ATG4B KO cells expressing GFP, WT-ATG4B or C74S-ATG4B respectively. Scale bar, 10 μm.

D. Proteolytic activity assay of recombinant His-ATG4B purified from *E. coli* using LC3B-mCherry substrate isolated from ATG4B knockout (KO) HEK293 cells. Immunoblot analysis shows cleavage of LC3B (detected by anti-mCherry and anti-LC3B antibodies) after incubation at 37°C for 30 minutes under the following conditions: (1) substrate-only control, (2) His-ATG4B + LC3-mCherry, and (3) His-ATG4B pre-treated with 100 μM N-ethylmaleimide (NEM). Arrowheads indicate processed LC3 (cleaved mCherry fragment) demonstrating cysteine protease-dependent activity.

E. Proteolytic cleavage of GST-PRMT1 by recombinant His-ATG4B. Purified His-ATG4B and GST-PRMT1 (both from *E. coli*) were mixed at the indicated ratios, incubated at 37°C for 30 minutes, and analyzed by immunoblotting with anti-GST and anti-PRMT1 antibodies. Cleavage products (cleaved PRMT1 and GST fragments) demonstrate dose-dependent processing, with asterisks (*) marking non-specific bands.

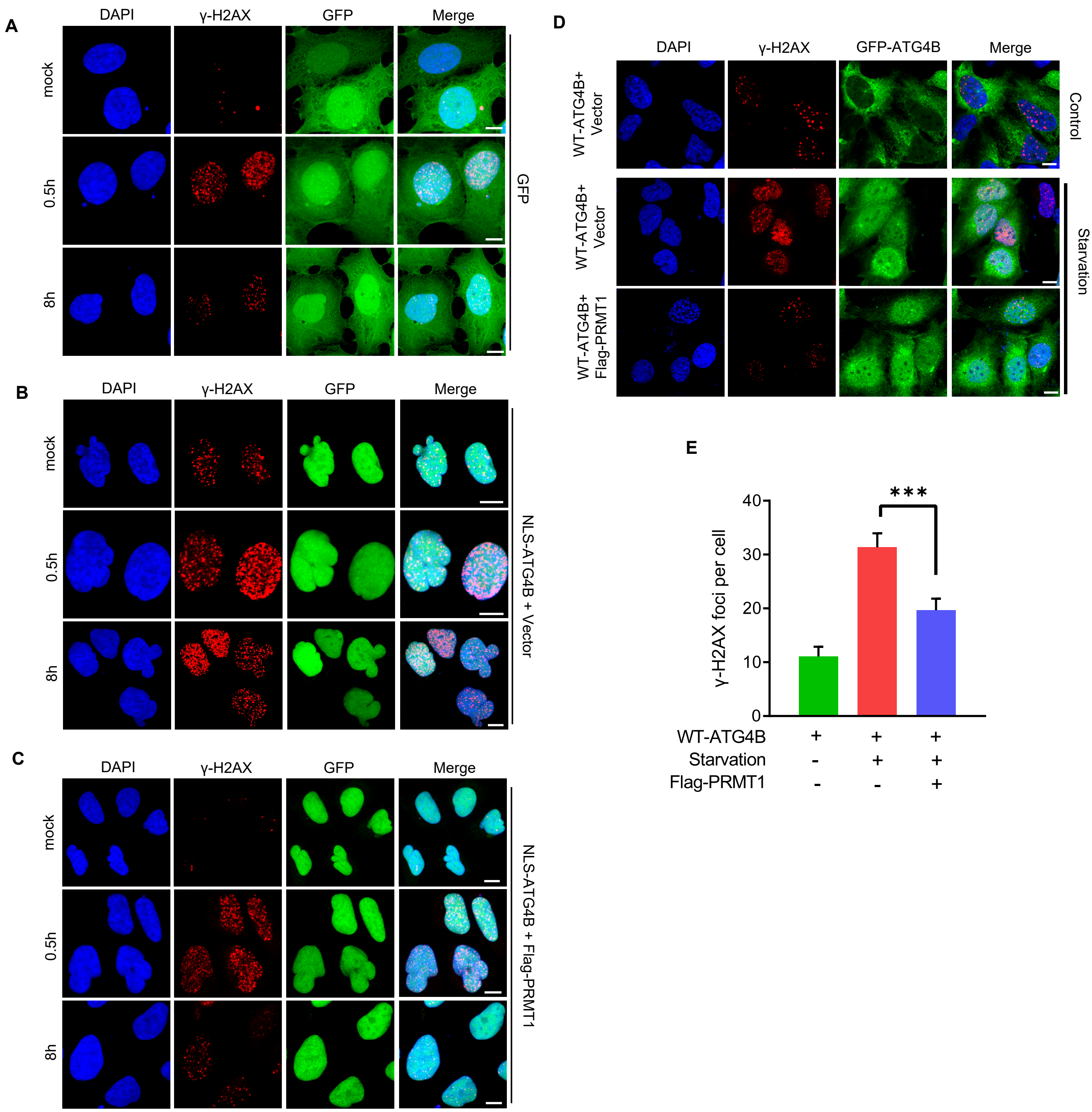

Supplementary Data Fig4. PRMT1 mediates the nuclear ATG4B-impaired DNA repair.

A-C. Representative images showing γ-H2AX foci after treated with 5μM Etoposide for 2 hours followed by recovery for indicated time (0.5 hours, 12 hours) in cells overexpressing GFP(**A**), NLS-ATG4B with Vector(**B**), or NLS-ATG4B with Flag-PRMT1(**C**) respectively. Scale bar, 10 μm.

D and E. Representative images(**D**) and quantification(**E**) of γ-H2AX foci under starvation or without treatment in WT-ATG4B cells overexpressing Vector or Flag-PRMT1 ($n \geq 50$ cells). Scale bar, 10μm. Data are represented as mean ± SEM, *** $p<0.001$.

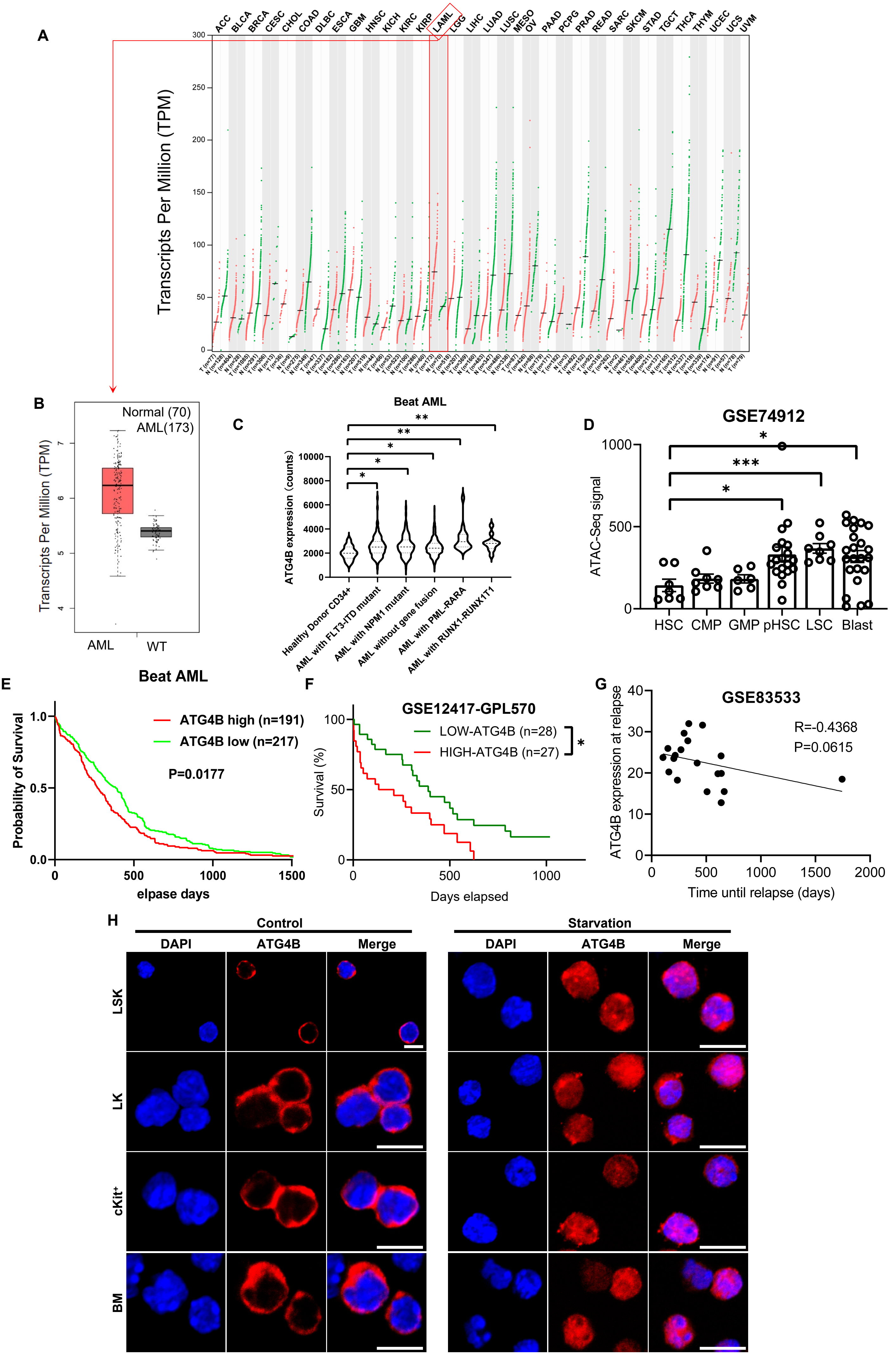

A
Transcripts Per Million (TPM)
ACC BLCA BRCA CESC CHOL COAD DLBC ESCA GBM HNSC KICH KIRC KIRP LAML LGG LIHC LUAD LUSC MESO OV PAAD PCPG PRAD READ SARC SKCM STAD TGCT THCA THYM UCEC UCS UVM
B
Normal (70)
AML(173)
Transcripts Per Million (TPM)
AML
WT
C
Beat AML
ATG4B expression (counts)
Healthy Donor CD34+
AML with FLT3-ITD mutant
AML with NPM1 mutant
AML without gene fusion
AML with PML-RARA
AML with RUNX1-RUNX1T1
D
GSE74912
ATAC-Seq signal
HSC CMP GMP pHSC LSC Blast
E
Beat AML
ATG4B high (n=191)
ATG4B low (n=217)
P=0.0177
Probability of Survival
elpase days
F
GSE12417-GPL570
LOW-ATG4B (n=28)
HIGH-ATG4B (n=27)
Survival (%)
Days elapsed
G
GSE83533
R=-0.4368
P=0.0615
ATG4B expression at relapse
Time until relapse (days)
H
Control
Starvation
DAPI
ATG4B
Merge
LSK
LK
cKit+
BM

Supplementary Data Fig 5. ATG4B highly expresses in acute myeloid leukemia and is associated with an unfavorable prognosis.

A. The expression of ATG4B in various tumor tissues and normal tissues from GEPIA databases.

B. ATG4B is highly expressed in acute myeloid leukemia (AML) samples (data are from GEPIA databases).

C. Violin plots showing ATG4B mRNA expression levels in distinct AML genetic subgroups from the Beat AML database, including Healthy donor $CD34^+$ (n=12), FLT3-ITD mutant (n=105), NPM1 mutant (n=108), cytogenetically normal (without recurrent fusion genes) (n=325), PML-RARA fusion-positive (n=15), and RUNX1-RUNX1T1 fusion-positive subtypes (n=11). Data represent normalized read counts. Statistical differences between groups were analyzed by two-tailed unpaired Student's t-test. * $p<0.05$, ** $p<0.01$.

D. Normalized ATAC-seq signal at the ATG4B locus in healthy donor-derived hematopoietic stem cells (HSCs) (n=7), common myeloid progenitors (CMPs) (n=8), granulocyte/macrophage progenitors (GMPs) (n=6), pre-leukemic HSCs (pHSCs) (n=19), leukemia stem cells (LSCs) (n=8), and leukemic blast cells (n=23). Each dot represents one individual AML patient sample. Data are presented as mean $\pm$ SEM. Statistical differences between groups were analyzed by two-tailed unpaired Student's t-test. * $p<0.05$, *** $p<0.001$.

E. Kaplan-Meier plots of overall survival in Beat AML cohorts for AML patients (n=408), stratified on the basis of ATG4B expression above (ATG4B high) or below (ATG4 Blow) the median. Data analyzed by the log-rank test.

F. Kaplan-Meier plots of overall survival in GSE12417-GPL570 cohorts for AML patients (n=55), stratified on the basis of ATG4B expression above (ATG4B high) or below (ATG4B low) the median. Data analyzed by the log-rank test. * $p<0.05$.

G. Pearson's correlation between ATG4B expression and days to relapse of AML patients (GSE83553; n = 19).

H. Representative images showing ATG4B subcellular distribution in LSK ($Lin^-cKit^+Sca1^+$), LK (Lin^-cKit^+), $cKit^+$ and bone marrow (BM) cells cultured in complete medium or starvation medium for 2 hours after sorted. Scale bar, 10μm.

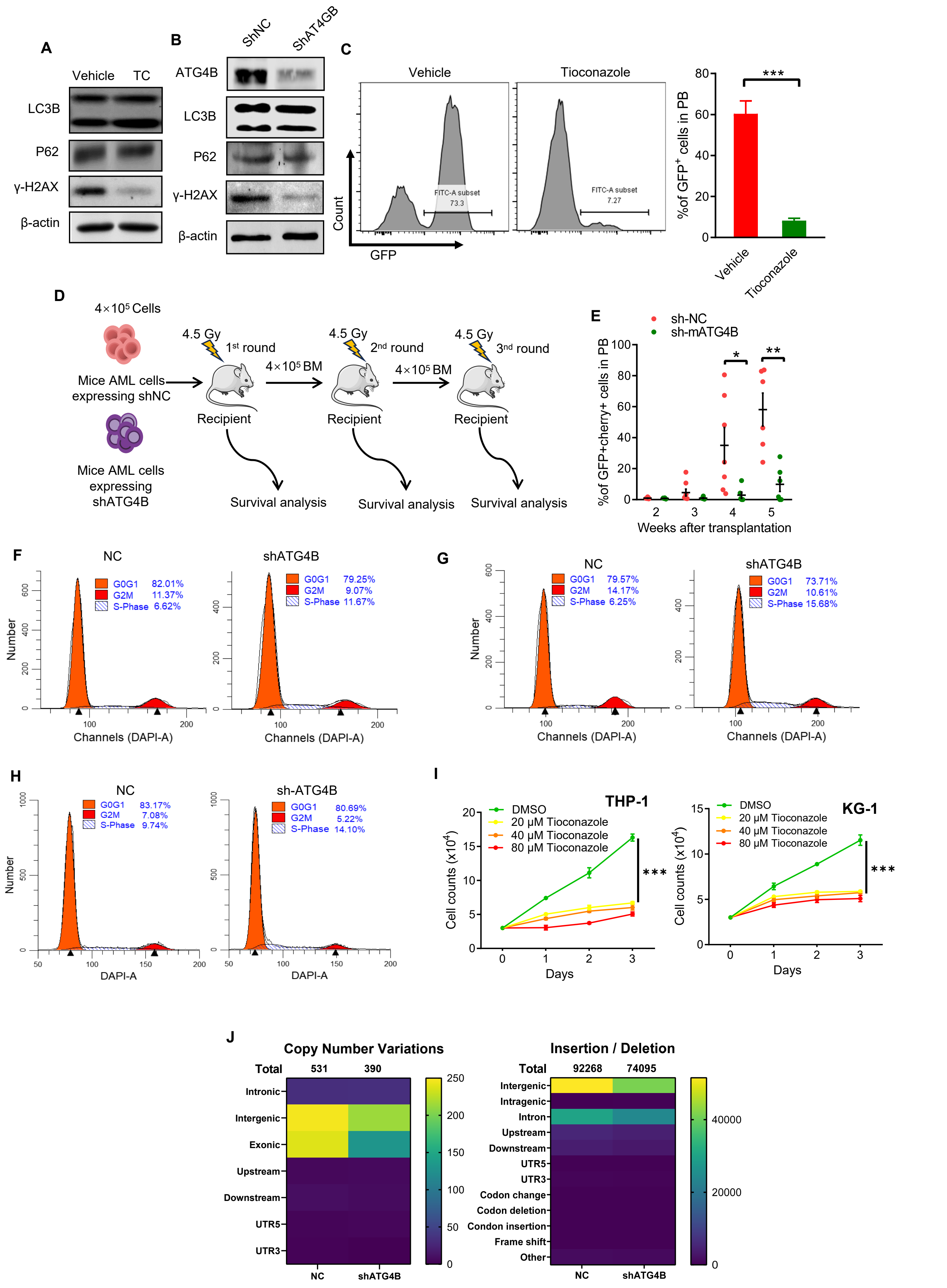

A
Vehicle TC
LC3B
P62
γ-H2AX
β-actin
B
ShNC ShAT4GB
ATG4B
LC3B
P62
γ-H2AX
β-actin
C
Vehicle
Tioconazole
Count
GFP
FITC-A subset 73.3
FITC-A subset 7.27
%of GFP+ cells in PB
***
Vehicle
Tioconazole
D
4×10⁵ Cells
Mice AML cells expressing shNC
Mice AML cells expressing shATG4B
4.5 Gy
1st round
2nd round
3rd round
4×10⁵ BM
Recipient
Survival analysis
E
sh-NC
sh-mATG4B
%of GFP+cherry+ cells in PB
Weeks after transplantation
F
NC
shATG4B
G0G1 82.01%
G2M 11.37%
S-Phase 6.62%
G0G1 79.25%
G2M 9.07%
S-Phase 11.67%
Number
Channels (DAPI-A)
G
NC
shATG4B
G0G1 79.57%
G2M 14.17%
S-Phase 6.25%
G0G1 73.71%
G2M 10.61%
S-Phase 15.68%
H
NC
sh-ATG4B
G0G1 83.17%
G2M 7.08%
S-Phase 9.74%
G0G1 80.69%
G2M 5.22%
S-Phase 14.10%
DAPI-A
I
THP-1
KG-1
DMSO
20 μM Tioconazole
40 μM Tioconazole
80 μM Tioconazole
Cell counts (x10⁴)
Days
***
J
Copy Number Variations
Total 531 390
Intronic
Intergenic
Exonic
Upstream
Downstream
UTR5
UTR3
NC shATG4B
Insertion / Deletion
Total 92268 74095
Intergenic
Intragenic
Intron
Upstream
Downstream
UTR5
UTR3
Codon change
Codon deletion
Condon insertion
Frame shift
Other

Supplementary Data Fig 6. ATG4B inhibition suppresses the progression of acute myeloid leukemia.

A. Western blot detecting LC3, p62 and γ-H2AX levels in bone marrow derived MLLT3-KMT2A+ AML cells treated with or without 40 μM Tioconazole.

B. Western blot detecting ATG4B, LC3, p62 and γ-H2AX levels in bone marrow derived MLLT3-KMT2A+ AML cells expressing ATG4B-targeted shRNA (shATG4B) or scrambled shRNA (shNC).

C. Representative FACS plots (Left) and the percentage (Right) of AML cells (GFP+) in peripheral blood of the AML mice intraperitoneally injected with 60mg/Kg Tioconazole or equivalent volume of vehicle every three days for 6 weeks (n = 6~8). Data are presented as mean ± SEM. Statistical differences between groups were analyzed by two-tailed unpaired Student's t-test. *** p<0.001.

D. Schematic diagram for the survival analysis of serial transplanting AML model mice. 4×10^5 mice bone marrow-derived AML cells expressing ATG4B-targeted shRNA (shATG4B) or scrambled shRNA (shNC) were transplanted into sub-lethally irradiated recipient mice (n = 15 per group). Next, 4×10^5 AML cells from the last round dying mice were used for the next round of transplantation. The survival curves of each round AML model mice were recorded.

E. Percentages of GFP+mCherry+ AML cells in peripheral blood of the recipient mice transplanted with AML cells (GFP+) expressing ATG4B targeted-shRNA (shATG4B, mCherry+) or scrambled shRNA (shNC, mCherry+) at the indicated time in the first transplantation (n = 6~8). Data are presented as mean ± SEM. Statistical differences between groups were analyzed by two-tailed unpaired Student's t-test. * p<0.05, ** p<0.01.

F-H. Representative images showing cell cycle ratio of bone marrow derived NC/shATG4B AML cells from 1st transplantation (**G**), 2nd transplantation (**H**) and 3rd transplantation (**I**) AML model mice.

I. Growth curves of THP-1 and KG-1 cells plated at 3×10^4 cells per well in 24-well plates and treated with DMSO or 20, 40, 80 μM Tioconazole (n=5 biological replicates), with cell counts quantified every 24 hours using trypan blue exclusion. Statistical significance was determined by two-way ANOVA with Tukey's multiple comparisons test to assess the effects of treatment concentration and time.

J. Heatmap visualization of copy number variations (CNVs, left) and insertion/deletion (InDel) densities (right) across chromosomal regions (exons, introns, and intergenic regions) in AML cells transduced with ATG4B-targeted shRNA (shATG4B) or scrambled shRNA (NC) , analyzed by whole-genome sequencing.
